# Genetic analysis of the cooperative tumorigenic effects of targeted deletions of tumor suppressors *Rb1*, *Trp53*, *Men1*, and *Pten* in neuroendocrine tumors in mice

**DOI:** 10.18632/oncotarget.27660

**Published:** 2020-07-14

**Authors:** Eugenia Y. Xu, Evan Vosburgh, Chung Wong, Laura H. Tang, Daniel A. Notterman

**Affiliations:** ^1^Rutgers Cancer Institute of New Jersey, Rutgers, The State University of New Jersey, New Brunswick, NJ 08903, USA; ^2^Department of Pediatrics, Robert Wood Johnson Medical School, Rutgers, The State University of New Jersey, New Brunswick, NJ 08901, USA; ^3^Department of Molecular Biology, Princeton University, Princeton, NJ 08544, USA; ^4^Department of Medicine, Veterans Administration Hospital, West Haven, CT 06516, USA; ^5^Department of Medicine, Yale University School of Medicine, New Haven, CT 06510, USA; ^6^Department of Pathology, Memorial Sloan-Kettering Cancer Center, New York, NY 10065, USA; ^7^Current address: Regeneron Inc., Tarrytown, NY 10591, USA

**Keywords:** neuroendocrine tumors, *RB1*, *Trp53*, *PTEN*, *Men1*

## Abstract

Genetic alterations of tumor suppressor genes (TSGs) are frequently observed to have cumulative or cooperative tumorigenic effects. We examined whether the TSGs *Rb1*, *Trp53*, *Pten* and *Men1* have cooperative effects in suppressing neuroendocrine tumors (NETs) in mice. We generated pairwise homozygous deletions of these four genes in insulin II gene expressing cells using the Cre-LoxP system. By monitoring growth and examining the histopathology of the pituitary (Pit) and pancreas (Pan) in these mice, we demonstrated that pRB had the strongest cooperative function with PTEN in suppressing PitNETs and had strong cooperative function with Menin and TRP53, respectively, in suppressing PitNETs and PanNETs. TRP53 had weak cooperative function with PTEN in suppressing pituitary lesions. We also found that deletion of *Pten* singly led to prolactinomas in female mice, and deletion of *Rb1* alone led to islet hyperplasia in pancreas. Collectively, our data indicated that pRB and PTEN pathways play significant roles in suppressing PitNETs, while the Menin-mediated pathway plays a significant role in suppressing PanNETs. Understanding the molecular mechanisms of these genes and pathways on NETs will help us understand the molecular mechanisms of neuroendocrine tumorigenesis and develop effective preclinical murine models for NET therapeutics to improve clinical outcomes in humans.

## INTRODUCTION

Human pituitary neuroendocrine tumors (PitNETs) are the third most common intracranial neoplasms and represent approximately 10–25% of all primary intracranial tumors [1, 2]. Pituitary carcinomas are highly aggressive and represent < 1% of pituitary tumors. The pituitary gland sits at the pituitary fossa and contains anterior, intermediate and posterior lobes. The anterior lobe mainly secretes prolactin, growth hormone (GH), adrenocorticotropin (ACTH), thyroid-stimulating hormone (TSH), and gonadotropin [follicle-stimulating hormone (FSH) and luteinizing hormone (LH)]. The posterior lobe is considered an extension of the hypothalamus and secretes oxytocin and anti-diuretic hormone (ADH). The intermediate lobe is located between anterior and posterior lobes. It secretes melanocyte-stimulating hormone (MSH) during fetal life but is small or absent in adults. Although PitNETs are generally benign monoclonal neoplasms, they can cause significant morbidity including visual disturbances caused by mass effects that lead to compression of adjacent structures, and/or deregulated hormone secretion, and then mortality [3, 4]. PitNETs can be characterized based on cell of origin and the types of hormone secreted, like prolactinomas (40–57%), nonfunctioning (15–37%), and ACTH-secreting (1.6–5.9%) [5–7]. Pituitary pathogenesis is challenging to study due to its unique biology and behavior. The molecular mechanism of tumor progression in the pituitary remains unclear.

Human pancreatic neuroendocrine neoplasms are classified as either well-differentiated (WD) pancreatic neuroendocrine tumors (PanNETs) or islet cell tumors and poorly differentiated (PD) pancreatic neuroendocrine carcinomas (PanNECs) [8, 9]. Based on Ki67 proliferation rate, WD-PanNETs are also classified as grade 1 (G1), grade 2 (G2), and grade 3 (G3) PanNETs with Ki67 index of < 3%, 3–20% and > 20%, respectively [9, 10]. PD-PanNECs are not derived from pancreatic islet cells; they are inevitably high grade with Ki67 index of > 20%. PanNETs are the second-most common pancreatic malignancy but only represent 1–2% of pancreatic tumors, and are far less aggressive than the most common pancreatic ductal adenocarcinoma. Even though less aggressive, PanNETs have limited treatment options if not resectable at diagnosis [11]. WD-PanNETs and PD-PanNECs develop as a result of different genetic alterations [12]. Understanding the molecular mechanism of tumorigenesis may aid in the development of novel therapeutic options.

Tumorigenesis is a multistep process involving alterations of oncogenes and/or tumor suppressor genes (TSGs) in a single cell. In contrast to oncogene activation, tumors resulting from TSG inactivation usually require both alleles to be lost, according to the Knudson’s “two-hit” hypothesis [13]. *TP53* and retinoblastoma susceptibility gene (*RB1*) encode classic tumor suppressors and are commonly inactivated or deregulated in human cancers [14–16]. Genetic mutations of *TP53* are observed in about 4% of PanNETs and the mutation rate for *RB1* appears to be very low. While the rates are low, mutations in both genes are often associated with aggressive PD-PanNECs [10]. Genetic mutations of *RB1* and *TP53* in human PitNETs are even less common [17–24]. Two studies have indicated that approximately 90% of PitNETs have at least one RB pathway gene silenced due to promoter methylation [25, 26]. Additionally, in rare cases *RB1* has been found with epigenetic mutations in the promoter region in PitNETs [27, 28] suggesting that inactivation of the RB pathway contributes to the development of PitNETs. *Rb1* mouse models develop highly penetrant pituitary tumors—ACTH-secreting tumors in most cases [29, 30], evidence that supports RB pathway playing a role in pituitary tumorigenesis. In mice, deletion of the *Trp53* gene leads to a wide spectrum of tumors but does not lead to NETs. However, deletion of *Trp53* accelerates NET development in *Rb1^+/–^* mice suggesting that TRP53 plays a role in NE tumorigenesis [31, 32]. Therefore, although *RB1* and *TP53* may not mutate frequently, both the TP53 and RB pathways are compromised in human NETs.

Menin is a 68 KDa protein encoded by the *MEN1* gene, a tumor suppressor gene mutated in Multiple Endocrine Neoplasia Type 1 (MEN1) [33]. MEN1 is an autosomal dominant tumor syndrome with high penetrance characterized by the presence of several endocrine tumors derived from pituitary, parathyroid, and pancreatic islet cells [34]. *MEN1* mutations are also observed in around 44% of non-familial human PanNETs, most often WD G1/G2 PanNETs [35, 36]. Several *Men1* mouse models generated by targeted mutation of the *Men1* gene [37–39] effectively mimic the tumor spectrum in humans. The PTEN (Phosphatase and TENsin homolog) tumor suppressor, a key negative regulator of the PI3K/AKT pathway encoding a lipid phosphatase, is located on a genomic region that frequently suffers loss of heterozygosity (LOH) in different types of advanced human cancers. Genetic mutations of *PTEN* are observed in about 7–26.4% of human PanNETs [35, 36, 40–45]. Reduced PTEN expression and increased PI3K/AKT pathway activity have been observed in human patients with pituitary tumors [46, 47]. Compound mice with concomitant deletions of *Men1* and *Pten* develop PitNETs and PanNETs and mice with *p18^–/–^ Pten^+/–^* mutations develop PitNETs [48, 49], suggesting that PTEN plays a role in pituitary and pancreatic islet tumorigenesis. But whether deletion of *Pten* alone induces PitNETs in mice is still unknown.

Tissue-specific homozygous deletion of TSGs in mice provides a powerful tool to understand the genetic basis of tumor progression. Functional cooperation between loss-of-function mutations targeting TSGs is commonly required for the progression of a normal cell into a cancerous one. We have investigated how pairwise deletions of TSGs cooperate in neuroendocrine tumorigenesis in mice. Double heterozygous *Men1* and *Rb1* knockout mice have been reported to develop the same tumor spectrum as the respective single knockout mice [50, 51]. The absence of cumulative effects from *Men1* and *Rb1* mutations leads to the suggestion that Menin and pRB are in the same molecular pathway of tumor suppression. However, *Men1* deletion mice develop *pars distalis* prolactinomas and *Rb1* deletion mice develop *pars intermedia* tumors of pituitary, suggesting that the functions of Menin and pRB may not fully overlap. Here we investigate the question of whether *Men1* and *Rb1* have cooperative tumorigenic effects on NETs using tissue-specific double homozygous deletions of *Men1* and *Rb1* in mice. To systematically test how deficiencies in the TSGs *Rb1*, *Men1*, *Trp53*, and *Pten* cooperate in tumorigenesis in mice, we have conditionally inactivated these genes in pairs in insulin II-expressing cells using the Cre-LoxP system in which Cre recombinase is under the control of Rat Insulin II gene Promoter (RIP-Cre). We used a targeted system as mouse models bearing complete *Rb1*, *Men1*, and *Pten* gene loss display embryonic lethality. We report here the characterization of PitNETs and PanNETs with double homozygous deletions of TSGs (Table 1) and illustrate that pRB has the strongest cooperative function with PTEN in suppressing PitNETs and has strong cooperative function with Menin and TRP53, respectively, in suppressing PitNETs and PanNETs in mice. Our data demonstrate that the pRB and PTEN pathways play significant roles in suppressing PitNETs while the Menin pathway plays a significant role in suppressing PanNETs in mice.

**Table 1:** Summary of the phenotypes of mice with various genotypes

Acronyms	Genotypes	Onset of Death^*^	Types of PitNETs^**^	Histology of pancreas/Onset of PanNETs^**^
MRbR	*Men1^flox/flox^ Rb1^flox/flox^* RIP-Cre	10 weeks	ACTH-secreting PitNETs	Hyperplasia to WD G1/G2 PanNETs/10 weeks
MRb	*Men1^flox/flox^ Rb1^flox/flox^*	None	NA	Normal /NA
MR	*Men1^flox/flox^* RIP-Cre	None	Prolactinomas	Hyperplasia to WD G1/G2 PanNETs/23 weeks
RbR	*Rb1^flox/flox^* RIP-Cre	16 weeks	ACTH-secreting PitNETs	Hyperplasia/NA
M”1”RbRC	*Men1^flox/+^ Rb1^flox/flox^* RIP-Cre	11 weeks	ACTH-secreting PitNETs	Hyperplasia/NA
MRb”1”RC	*Men1^flox/flox^ Rb1^flox/+^* RIP-Cre	27 weeks	Prolactinomas	Hyperplasia to PanNETs/NA
M”1”Rb”1”RC	*Men1^flox/+^ Rb1^flox/+^* RIP-Cre	None	NA	Normal/NA
PR	*Pten^flox/flox^* RIP-Cre	None	Prolactinomas	Hyperplasia/NA
P	*Pten^flox/flox^*	None	NA	Normal/NA
M”1”PRC	*Men1^flox/+^ Pten^flox/flox^* RIP-Cre	15 weeks	Prolactinomas	Hyperplasia/NA
MP”1”RC	*Men1^flox/flox^ Pten^flox/+^* RIP-Cre	23 weeks	Prolactinomas	Hyperplasia to PanNETs/NA
M”1”P”1”RC	*Men1^flox/+^ Pten^flox/+^* RIP-Cre	None	NA	Normal/NA
PRbR	*Pten^flox/flox^ Rb1^flox/flox^* RIP-Cre	4 weeks	ACTH-secreting PitNETs	Normal/NA
PRb	*Pten^flox/flox^ Rb1^flox/flox^*	None	NA	Normal/NA
53PR	*Trp53^flox/flox^ Pten^flox/flox^* RIP-Cre	17 weeks	Enlarged pituitary	Hyperplasia/NA
53P	*Trp53^flox/flox^ Pten^flox/flox^*	None	NA	Normal/NA
53RbR	*Trp53^flox/flox^ Rb1^flox/flox^* RIP-Cre	9 weeks	ACTH-secreting PitNETs	Hyperplasia to G3 PanNETs/9 weeks
53Rb	*Trp53^flox/flox^ Rb1^flox/flox^*	None	NA	Normal/NA

Notes: ^*^None, no death during the study period; ^**^NA, not available.

## RESULTS

### *Rb1* and *Men1* function cooperatively to accelerate PitNETs and death

**Figure 1:**
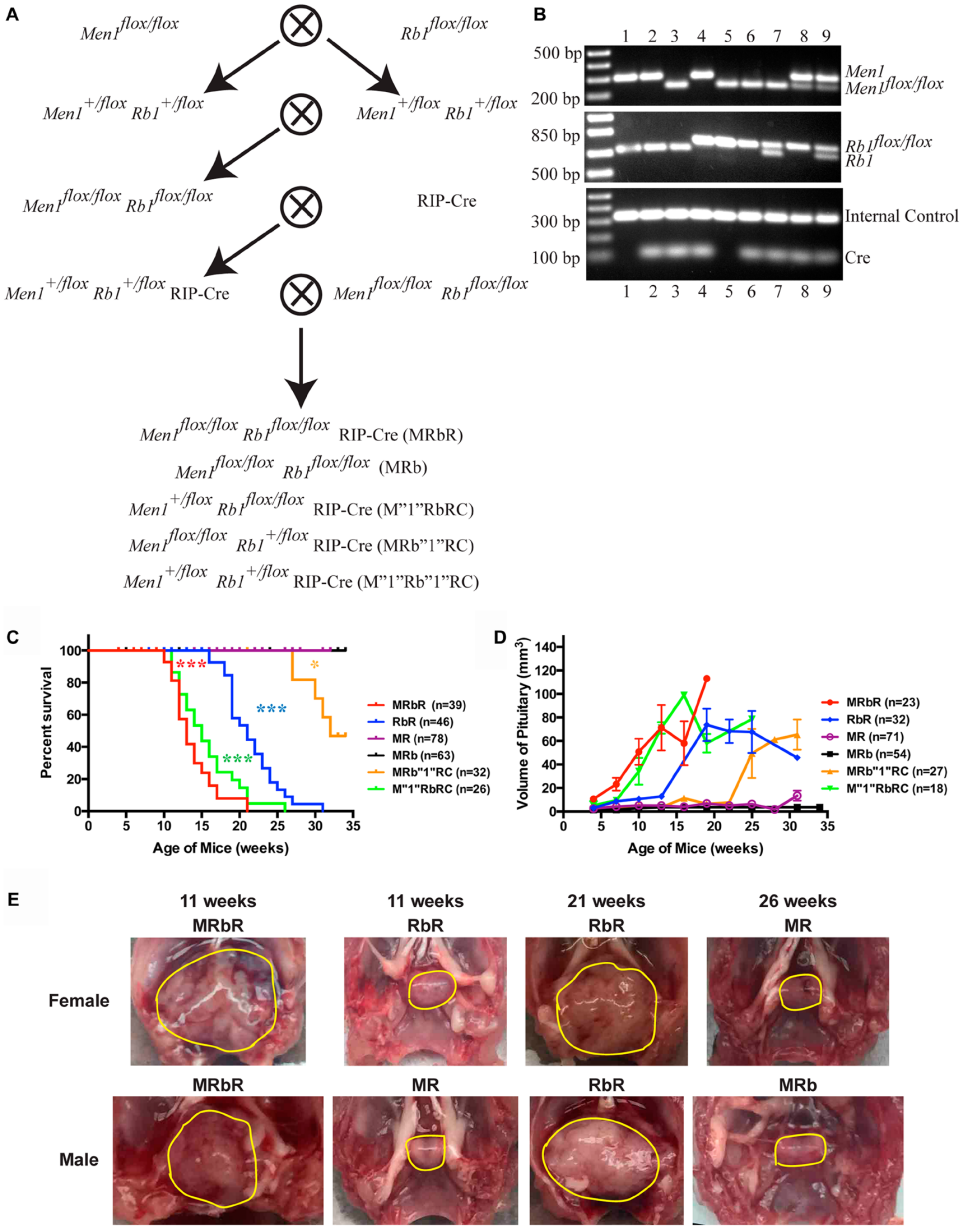
Concomitant loss of *Men1* and *Rb1* decreased survival and accelerated PitNET development in MRbR mice. (**A**) Diagram of the strategy used to generate compound mice MRbR and littermates MRb, M”1”RbRC, MRb”1”RC, and M”1”Rb”1”RC. (**B**) Representative genotyping results of the litters in A by PCR analysis using tail genomic DNA. Genotypes of each lane: 1-WT, 2-RIP-Cre, 3-*Men1^flox/flox^* RIP-Cre (MR), 4-*Rb1^flox/flox^* RIP-Cre (RbR), 5-*Men1^flox/flox^ Rb1^flox/flox^* (MRb), 6-*Men1^flox/flox^ Rb1^flox/flox^* RIP-Cre (MRbR), 7-*Men1^flox/flox^ Rb1^+/flox^* RIP-Cre (MRb”1”RC), 8-*Men1^+/flox^ Rb1^flox/flox^* RIP-Cre (M”1”RbRC), and 9-*Men1^+/flox^ Rb1^+/flox^* RIP-Cre (M”1”Rb”1”RC). (**C**) Kaplan–Meier survival curves showed significantly shorter life spans (*p* < 0.0001) in double deletions MRbR mice than single deletion MR, RbR, and corresponding wild-type control MRb mice; The survival curve of MRbR mice had no significant difference from that of M”1”RbRC mice. M”1”RbRC mice showed significantly shorter life spans than RbR mice (*p* < 0.0001), and MRb”1”RC mice (*p* < 0.0001). MRb”1”RC mice showed shorter life spans than MR mice (*p* < 0.0176). (**D**) Evaluation of the sizes of pituitaries in three-week intervals starting at 4 weeks in double deletions MRbR mice and their littermates, single deletion MR and RbR mice at scheduled autopsy showed that the pace of pituitary growth is consistent with the pattern of the survival curves in C. (**E**) Gross pathology of pituitary shown from representative MRbR, RbR, MR, and MRb female and male mice at specified age. Normal pituitary is cylindrical in shape as seen in wild-type control MRb mice. Pituitaries or PitNETs were circled in yellow lines inside the mouse skull. ^***^ *p*-value < 0.0001; ^*^ *p*-value < 0.05.

To investigate whether Menin and pRB function cooperatively in neuroendocrine tumorigenesis, we generated compound *Men1^flox/flox^ Rb1^flox/flox^* RIP-Cre (MRbR) mice with concomitant homozygous deletions of *Men1* and *Rb1* in the pancreas and pituitary through a series of crosses (Figure 1A). Mice with other genetic combinations generated from these series of crosses were examined as well. The genotypes of the compound mice were confirmed by PCR analysis using genomic tail DNA (Figure 1B). We monitored the survival of a cohort of double homozygous deletions MRbR mice, alongside control mice—wild-type control *Men1^flox/flox^ Rb1^flox/flox^* (MRb) without RIP-Cre transgene, the single homozygous deletion *Men1^flox/flox^* RIP-Cre (MR) and *Rb1^flox/flox^* RIP-Cre (RbR) (Figure 1C). Wild-type control MRb and single deletion MR mice were viable during the study period of thirty-four weeks, as previously reported for MR mice [37]; Single deletion RbR mice started dying at sixteen weeks, had a median survival of twenty-one weeks and did not live beyond thirty-one weeks; double homozygous deletions MRbR mice started dying at ten weeks, had a median survival of thirteen weeks and did not live beyond twenty-one weeks. These data indicated that concomitant loss of *Rb1* and *Men1* accelerated death (*p* < 0.0001) more than a single deletion; and *Rb1* deletion alone had a more severe effect on survival than *Men1* deletion alone (*p* < 0.0001).

To understand the cause of death of these mice, the sick mice were autopsied. PitNETs were observed in both MRbR, and RbR mice (Figure 1E). The nineteen sick MRbR mice (9F/10M) and the twenty-three sick RbR mice (8F/15M) all showed symptoms such as loss of vision, tilted head/body—symptoms consistent with those described in human patients with PitNETs and as reported in *Men1^flox/flox^ Pten^flox/flox^* RIP-Cre (MPR) mice [48]. Wild-type control MRb and single deletion MR mice of the same age displayed normal or slightly enlarged pituitaries, respectively. Evaluation of the pituitary size in a cohort of MRbR, RbR, MR, and MRb mice as they age displayed that pituitaries grew fastest in MRbR mice (Figure 1D). RbR mice grew dramatically faster and bigger pituitaries than MR and wild-type control MRb mice. Death of MRbR and RbR mice was due to PitNETs. Western blot analysis confirmed that Menin and/or RB1 expression was knocked down in the pituitary in representative MRbR, MR, and RbR mice (data not shown). Concomitant loss of *Rb1* and *Men1* in mice resulted in an earlier onset of PitNETs compared to single deletion of *Men1* and *Rb1*, suggesting that pRB and Menin function cooperatively to suppress pituitary tumorigenesis. Earlier onset of PitNETs and death in RbR mice than MR mice suggested that pRB plays a more significant role in suppressing pituitary tumorigenesis compared to Menin.

**Figure 2:**
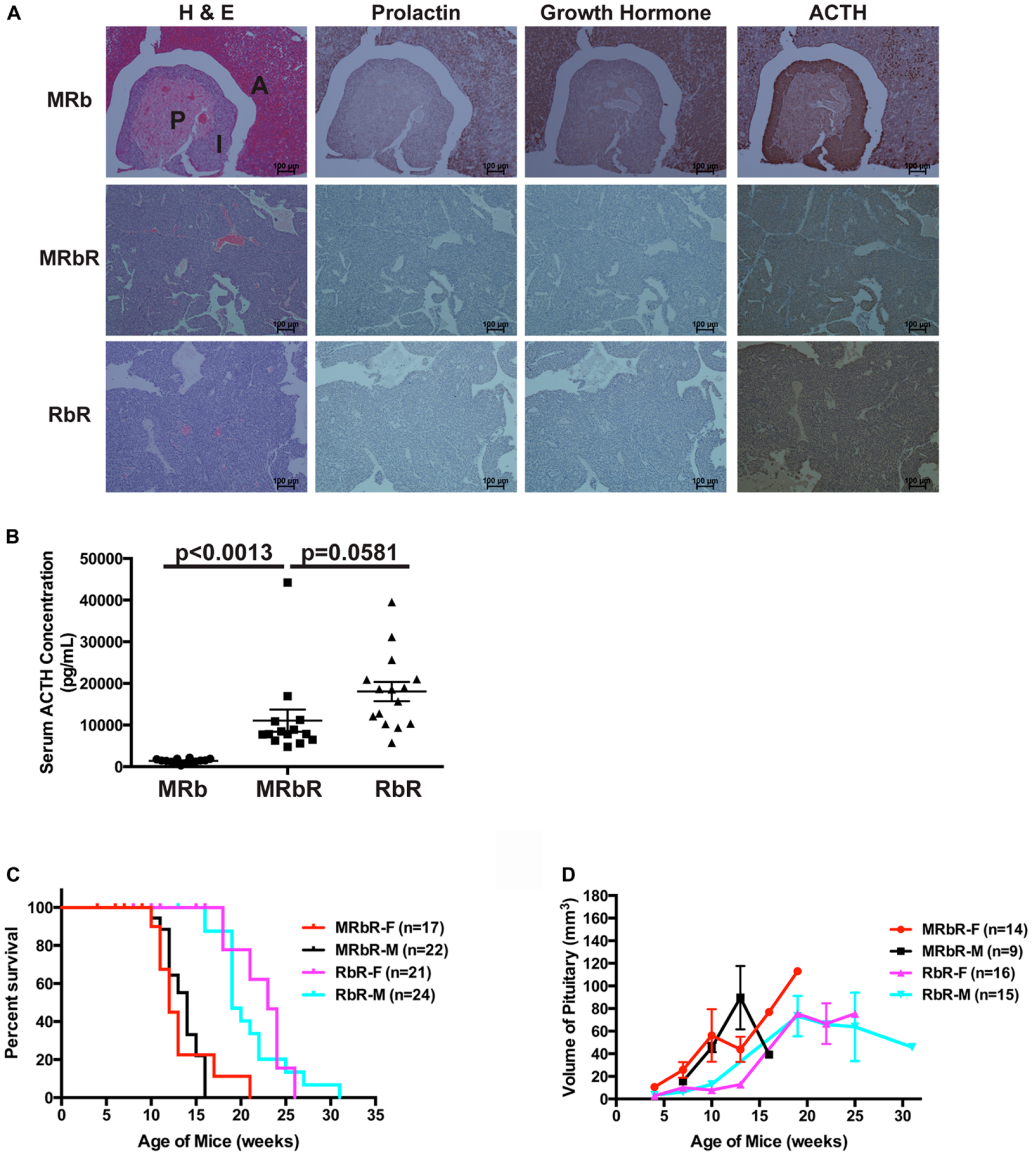
MRbR mice developed ACTH-secreting PitNETs. (**A**) H & E, IHC staining for prolactin, growth hormone, and ACTH on pituitary sections in MRb, MRbR, and RbR mice. Anterior lobe (A), Intermediate lobe (I), and Posterior lobe (P) of normal pituitary in MRb mice are shown in the H & E section. (**B**) Serum ACTH levels using ELISA assays in MRb, MRbR, and RbR mice confirmed that the PitNETs from MRbR and RbR mice were ACTH-secreting tumors. Serum ACTH levels between MRbR and RbR mice were not significantly different (*p* = 0.0581), but were significantly higher than that in MRb mice of the same age and sex as that of MRbR mice (*p* < 0.0013). (**C** and **D**) No gender bias was observed in MRbR and RbR mice for survival or pituitary growth as they age. (C) Survival curve. The survival curves of MRbR (*p* = 0.6612) and RbR (*p* = 0.7535) mice showed no statistical significances between female and male mice. (D) Pituitary growth.

To understand the pituitary origin of these tumors, the PitNETs from MRbR (*n* = 11) and RbR (*n* = 8) mice and the pituitaries from wild-type control MRb (*n* = 11) mice were immunohistochemically stained for prolactin, GH, and ACTH. MRb mice showed staining consistent with a normal pituitary–heterogeneous staining of prolactin, GH, and ACTH in the anterior lobe; negative staining of prolactin and GH in the intermediate and posterior lobes; positive ACTH staining in the intermediate lobe but negative staining in the posterior lobe, which is as reported, ACTH-secreting corticotrophs represent the major cell type in the pars intermedia in mice [52]. PitNETs from MRbR and RbR mice showed negative staining of prolactin and GH, but positive staining of ACTH (Figure 2A). Serum ELISA assays confirmed the ACTH-secreting PitNETs (Figure 2B, prolactin and GH results not shown). RbR mice showed variable levels of serum ACTH concentration because RbR mice developed PitNETs at a wider age range than MRbR mice. These PitNETs arose from the intermediate lobe, as reported in *Rb1* mutation mice [53]. Consistent with ACTH-secreting PitNETs, MRbR (*p* = 0.6612), and RbR (*p* = 0.7535) mice did not show gender bias in death and pituitary size as mice aged (Figure 2C and 2D). The PitNETs developed in MRbR mice were attributable to the loss of pRb, supporting that pRB plays a significant role in suppressing pituitary tumorigenesis compared to Menin.

### *Rb1* and *Men1* function cooperatively to accelerate PanNETs

**Figure 3:**
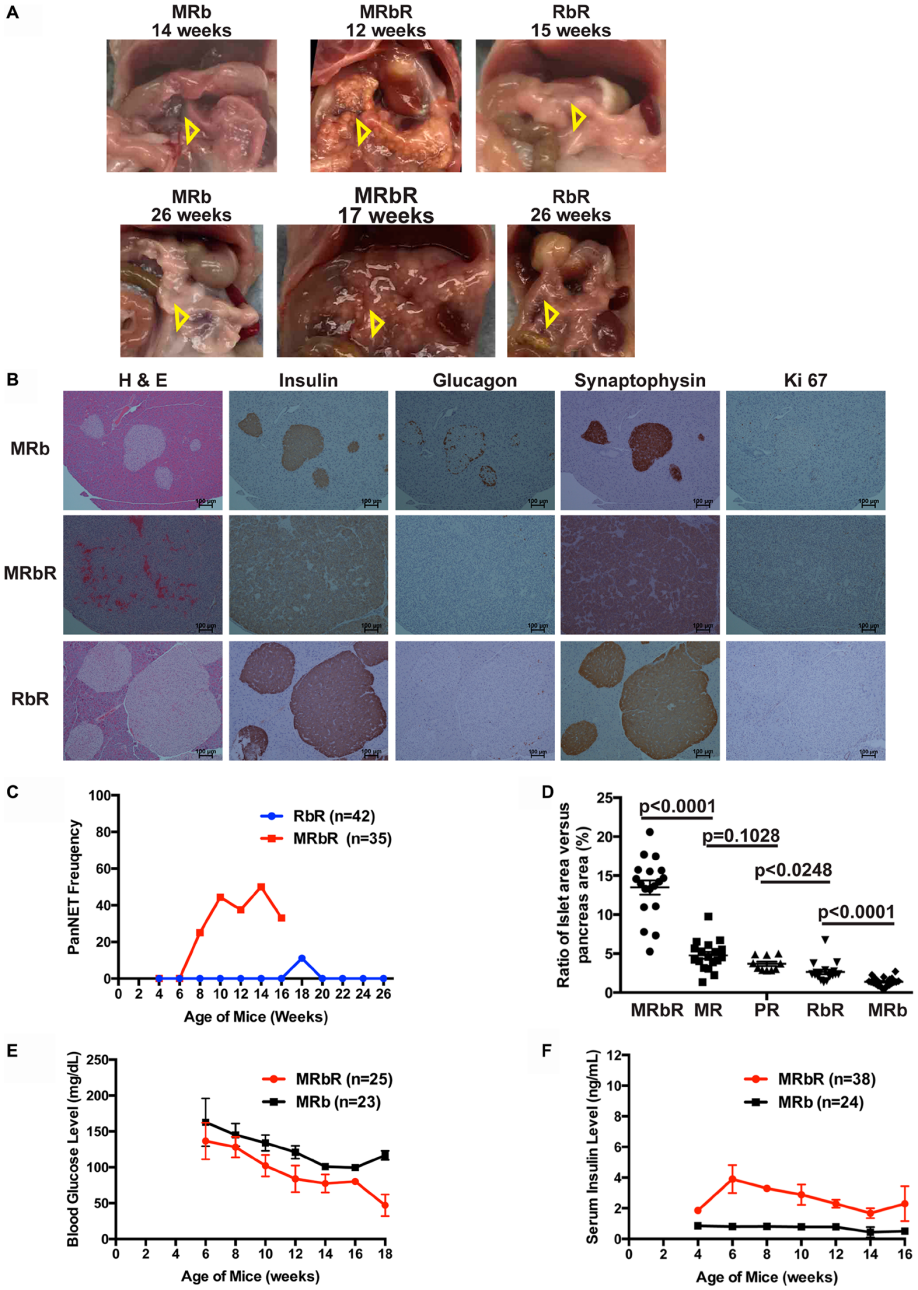
Concomitant loss of *Men1* and *Rb1* accelerated PanNET development in MRbR mice. (**A**) Gross pathology of pancreas in MRb, MRbR and RbR mice at the specified age. Pancreas was shown with open triangle inside the mouse abdomen. (**B**) H & E, IHC staining of Insulin, Glucagon, Synaptophysin and Ki-67 of MRb, MRbR and RbR pancreas sections. (**C**) Frequency of PanNETs in MRbR and RbR mice at scheduled autopsy. (**D**) Quantitative comparison of the ratio of the islets area per pancreas area from double deletions MRbR, single deletion MR, PR, and RbR, and wild-type control MRb mice of 15–17 weeks with shown *p*-values. (**E**) Blood glucose level in MRbR and MRb mice as they age (**F**) Serum insulin level in MRbR and MRb mice as they age.

We next investigated the effect of the concomitant loss of *Rb1* and *Men1* in the pancreas in comparison with the effect of single *Men1* or *Rb1* deletion. We evaluated the pancreas of double deletions MRbR (*n* = 36) and single deletion RbR (*n* = 46) mice along with wild-type control MRb (*n* = 62) and single deletion MR (*n* = 78) mice macroscopically and evaluated the histopathology of the pancreas in a subset of the mice. Wild-type control MRb mice showed normal pancreas both macroscopically and microscopically with few small, round islets with normal distribution of α cells (Figure 3A and 3B). Macroscopic examination of pancreas in RbR mice showed normal and abnormal pancreas. In evaluating the histology of pancreas sections from RbR mice (*n* = 42), only one RbR mouse pancreas at eighteen weeks (*n* = 20 mice between age 18–31 weeks) showed tumor and the rest only showed hyperplasia (Figure 3B and 3C). Almost all RbR mice developed hyperplasia without pancreatic tumors in pancreas but all sick RbR mice developed PitNETs in brain, supporting that death of RbR mice was due to development of PitNETs (Table 2). MR mice showed PanNETs after twenty-three weeks and with increasing penetrance as mice aged (data not shown [37]), indicating that deletion of *Rb1* had less effect on islet tumorigenesis than deletion of *Men1*.

**Table 2:** Histology of pancreas, size of pituitary and health condition in RbR mice

Age of Mice (weeks)	Mouse Number	Histology of Pancreatic islets	Size of pituitary (L × W × H mm)^*^	Mouse health condition
4	RbR mouse 1	Normal/ hyperplasia	3 × 1 × 1	Healthy
4	RbR mouse 2	Normal/ hyperplasia	3 × 1 × 1	Healthy
4	RbR mouse 3	Normal/ hyperplasia	3 × 1 × 1	Healthy
6	RbR mouse 4	Normal/ hyperplasia	4 × 2 × 1.5	Healthy
6	RbR mouse 5	Normal/ hyperplasia	3 × 1.5 × 1.5	Healthy
6	RbR mouse 6	Normal/ hyperplasia	3 × 2 × 2	Healthy
8	RbR mouse 7	Normal/ hyperplasia	4 × 2.5 × 2	Healthy
8	RbR mouse 8	Hyperplasia	3 × 2 × 2	Healthy
10	RbR mouse 9	Hyperplasia	3 × 2 × 2	Healthy
10	RbR mouse 10	Normal/ hyperplasia	4 × 2 × 2	Healthy
10	RbR mouse 11	Hyperplasia	4 × 2 × 2	Healthy
10	RbR mouse 12	Hyperplasia	4 × 2 × 2	Healthy
10	RbR mouse 13	Hyperplasia	5 × 4 × 2	Healthy
10	RbR mouse 14	Hyperplasia	4.5 × 3.5 × 2	Healthy
10	RbR mouse 15	Hyperplasia	3 × 2 × 2	Healthy
10	RbR mouse 16	Hyperplasia	4 × 3.5 ×2	Healthy
10	RbR mouse 17	Hyperplasia	3 × 2 × 2	Healthy
12	RbR mouse 18	Hyperplasia	NA	Healthy
14	RbR mouse 19	Normal/ hyperplasia	3.5 × 3.5 × 2	Healthy
16	RbR mouse 20	Normal/ hyperplasia	NA	Healthy
16	RbR mouse 21	Normal/ hyperplasia	NA	Healthy
16	RbR mouse 22	Normal/ hyperplasia	NA	Sick
18	RbR mouse 23	Hyperplasia	NA	Healthy
18	RbR mouse 24	Hyperplasia	NA	Healthy
18	RbR mouse 25	Tumor	NA	Sick
18	RbR mouse 26	Hyperplasia	NA	Sick
18	RbR mouse 27	Hyperplasia	7.5 × 5 × 2.5	Sick
18	RbR mouse 28	Hyperplasia	8 × 5 × 2.5	Sick
18	RbR mouse 29	Hyperplasia	NA	Sick
18	RbR mouse 30	Normal/ hyperplasia	NA	Sick
18	RbR mouse 31	Hyperplasia	NA	Sick
20	RbR mouse 32	Hyperplasia	7 × 6 × 3	Sick
20	RbR mouse 33	Hyperplasia	10 × 8 × 3	Sick
20	RbR mouse 34	Hyperplasia	8 × 6 × 3	Sick
22	RbR mouse 35	Hyperplasia	7 × 6 × 3	Sick
22	RbR mouse 36	Normal/ hyperplasia	8 × 6 × 3	Sick
22	RbR mouse 37	Hyperplasia	5 × 5 × 3	Sick
24	RbR mouse 38	Hyperplasia	10 × 6 × 3	Sick
24	RbR mouse 39	Hyperplasia	8 × 7.5 × 3	Sick
26	RbR mouse 40	Hyperplasia	8 × 6 × 3	Sick
27	RbR mouse 41	Hyperplasia	8 × 4 × 2	Sick
31	RbR mouse 42	Hyperplasia	7 × 5 × 2.5	Sick

Note: ^*^NA, not available (i.e., the size of pituitary was not measured).

Macroscopic examination of pancreas in double deletion MRbR mice demonstrated multifocal nodules after ten weeks in some mice (Figure 3A). Evaluation of the histology of pancreas sections in MRbR (*n* = 35) mice indicated that pancreatic tumors developed as early as eight to nine weeks and reached around 50% of mice by ten weeks (Figure 3B and 3C), much earlier than in mice with a single deletion of *Men1* or *Rb1*. MRbR mice with pancreatic tumors may not be sick but all sick mice developed PitNETs, supporting that death of MRbR mice was due to development of PitNETs (Table 3). The ratio of islets area per pancreas area in mice from fifteen to seventeen weeks was significantly greater for MRbR mice (*p* < 0.0001) compared to single deletion MR mice who had significantly more islets area per pancreas area than single deletion RbR mice (*p* < 0.0003) who had significantly more islets area per pancreas area than wild-type control MRb mice (Figure 3D). Collectively, concomitant deletion of *Men1* and *Rb1* accelerated tumor development in pancreas. Menin plays a more significant role in suppressing islet tumorigenesis than pRB.

**Table 3:** Histology of pancreas, size of pituitary and health condition in MRbR mice

Age of Mice (weeks)	Mouse Number	Histology of Pancreatic islets	Size of pituitary (L × W × H mm)	Mouse health condition
4	MRbR mouse 1	Hyperplasia	3.5 × 2 × 1.5	Healthy
6	MRbR mouse 2	Hyperplasia	5 × 3 × 1.5	Healthy
6	MRbR mouse 3	Hyperplasia	NA	Healthy
6	MRbR mouse 4	Hyperplasia	4 × 3.5 × 2	Healthy
6	MRbR mouse 5	Hyperplasia	NA	Healthy
6	MRbR mouse 6	Hyperplasia	5 × 3 × 1.5	Healthy
6	MRbR mouse 7	Hyperplasia	4 × 3 × 2.5	Healthy
8	MRbR mouse 8	Hyperplasia	5 × 5 × 2.5	Healthy
8	MRbR mouse 9	Hyperplasia	5 × 5 × 2.5	Healthy
8	MRbR mouse 10	Hyperplasia	NA	Healthy
8	MRbR mouse 11	Tumor	NA	Healthy
10	MRbR mouse 12	Hyperplasia	5 × 5 × 2.5	Healthy
10	MRbR mouse 13	Hyperplasia	NA	Sick
10	MRbR mouse 14	Tumor	NA	Healthy
10	MRbR mouse 15	Tumor	NA	Healthy
10	MRbR mouse 16	Tumor	NA	Healthy
10	MRbR mouse 17	Hyperplasia	6 × 6 × 2.5	Healthy
10	MRbR mouse 18	Hyperplasia	6 × 5 × 2.5	Healthy
10	MRbR mouse 19	Hyperplasia	10 × 8 × 3	Sick
10	MRbR mouse 20	Tumor	5 × 5 × 3	Sick
12	MRbR mouse 21	Hyperplasia	NA	Healthy
12	MRbR mouse 22	Hyperplasia	6 × 5 × 3	Sick
12	MRbR mouse 23	Tumor	NA	Sick
12	MRbR mouse 24	Hyperplasia	5 × 5 × 3	Sick
12	MRbR mouse 25	Hyperplasia	5 × 3.5 × 3	Sick
12	MRbR mouse 26	Tumor	NA	Sick
12	MRbR mouse 27	Hyperplasia	NA	Healthy
12	MRbR mouse 28	Tumor	5 × 5 × 3	Sick
14	MRbR mouse 29	Tumor	10 × 7.5 × 3	Sick
14	MRbR mouse 30	Tumor	7 × 5 × 3	Sick
14	MRbR mouse 31	Hyperplasia	8 × 4 × 2	Sick
14	MRbR mouse 32	Hyperplasia	5 × 5 × 2.5	Healthy
16	MRbR mouse 33	Hyperplasia	5 × 5 × 3	Sick
16	MRbR mouse 34	Hyperplasia	7 × 7 × 3	Sick
16	MRbR mouse 35	Tumor	12 × 6 × 3	Sick

Note: ^*^NA, not available (i.e., the size of pituitary was not measured).

MRbR pancreatic tumors displayed immunoreactivity to insulin, and the neuroendocrine markers chromogranin A (data not shown) and synaptophysin, indicating that these tumors were PanNETs (Figure 3B). The Ki-67 index of MRbR tumors was 2.8% (*n* = 5), specifying these were WD G1/G2 PanNETs. MRbR mice displayed lower blood glucose levels and higher serum insulin levels compared to control MRb mice of the same age (Figure 3E and 3F), indicating these PanNETs were insulinomas.

### *Rb1* plays a more significant role than Men1 in suppressing PitNETs

We compared pituitary tumorigenesis in mice with concomitant heterozygous deletion of *Men1* and homozygous deletion of *Rb1* (M”1”RbRC), concomitant homozygous deletion of *Men1* and heterozygous deletion of *Rb1* (MRb”1”RC), concomitant heterozygous deletion of *Men1* and heterozygous deletion of *Rb1* (M”1”Rb”1”RC). Survival, pathology and histology of these mice up to thirty-five weeks demonstrated: double heterozygous M”1”Rb”1”RC mice were viable and healthy during the study period and M”1”RbRC mice showed significantly shorter life spans than MRb”1”RC mice (*p* < 0.0001) (Figure 1C). The median survival of M”1”RbRC mice was fifteen weeks and that of MRb”1”RC mice was thirty-two weeks. M”1”RbRC mice had significantly shorter life spans than single deletion RbR mice (*p* < 0.0001) and MRb”1”RC mice had significantly shorter life spans than single deletion MR mice (*p* < 0.0176), indicating that suppression of death by pRB and Menin was dosage-dependent.

**Figure 4:**
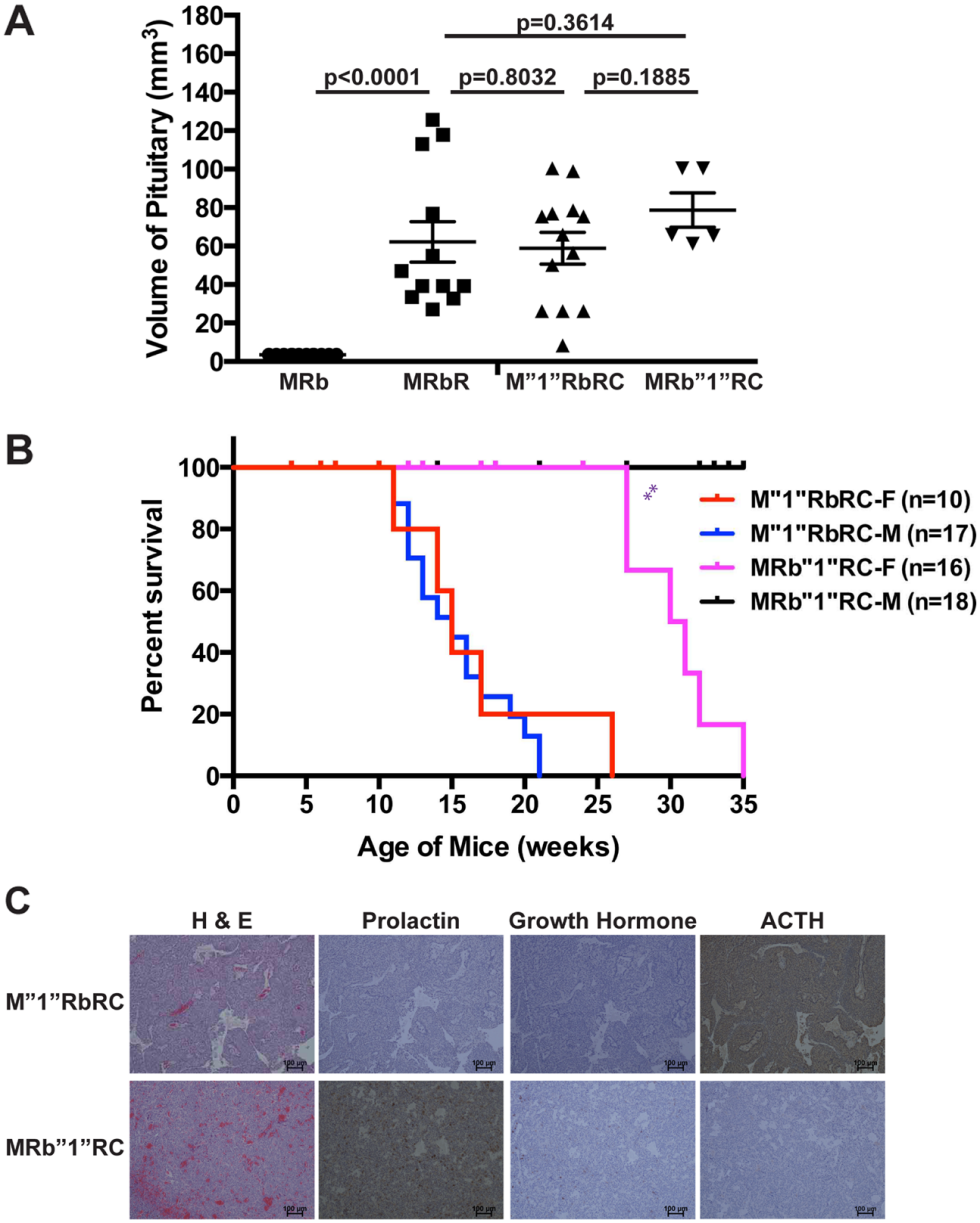
TSG *Rb1* plays a more significant role in suppressing PitNETs compared to *Men1*. (**A**) The sizes of PitNETs in MRbR, M”1”RbRC and MRb”1”RC at death were not significantly different from each other as shown *p*-values. Wild-type control mice MRb that were sex and age matched to double deletions MRbR mice showed normal pituitary. (**B**) Gender bias in survival was observed in MRb”1”RC mice (*p* < 0.0016) but was not observed in M”1”RbRC mice (*p* = 0.5267). (**C**) H & E, IHC staining of prolactin, growth hormone, and ACTH showed that M”1”RbRC mice developed ACTH-secreting PitNETs, while MRb”1”RC mice developed prolactinomas. ^**^ *p*-value < 0.01.

Autopsies of the sick mice displayed large PitNETs in all brains, and normal or abnormal pancreas, indicating that M”1”RbRC and MRb”1”RC mice were sick due to development of PitNETs (Figure 4A). The sizes of PitNETs at death were not significantly different between M”1”RbRC and MRb”1”RC mice (*p* = 0.1885), as well as not significantly different from MRbR mice (*p* = 0.8032 and *p* = 0.3614) respectively. Wild-type control MRb mice of the same age and sex as MRbR mice showed normal pituitary. The survival curve between M”1”RbRC and MRbR mice had no significant difference (*p* = 0.1325) (Figure 1C). However, the survival curve between MRbR mice and MRb”1”RC mice was significantly different (*p* < 0.0001). The growth pace of the pituitaries as mice aged was consistent with the pattern of their survival curves, suggesting that faster growth of PitNETs of these mice led to shorter life spans (Figure 1D) and indicating that deletion of *Men1* had less effect on pituitary tumorigenesis than deletion of *Rb1*.

Consistent with RbR mice developing ACTH-secreting PitNETs and MR mice developing prolactinomas, M”1”RbRC mice (*n* = 8) developed ACTH-secreting PitNETs and MRb”1”RC mice (*n* = 8) developed prolactinomas (Figure 4C). Further, female MRb”1”RC mice showed significantly shorter life spans (*p* < 0.0016) than male MRb”1”RC mice while female M”1”RbRC had similar life spans (*p* = 0.5267) to male M”1”RbRC mice, confirming a gender bias in mice developing prolactinomas (Figure 4B). Collectively, the suppression of PitNETs by pRB and Menin was dosage-dependent and pRB plays a more significant role in suppressing PitNETs over Menin.

### *Pten* deletion alone led to PitNETs

**Figure 5:**
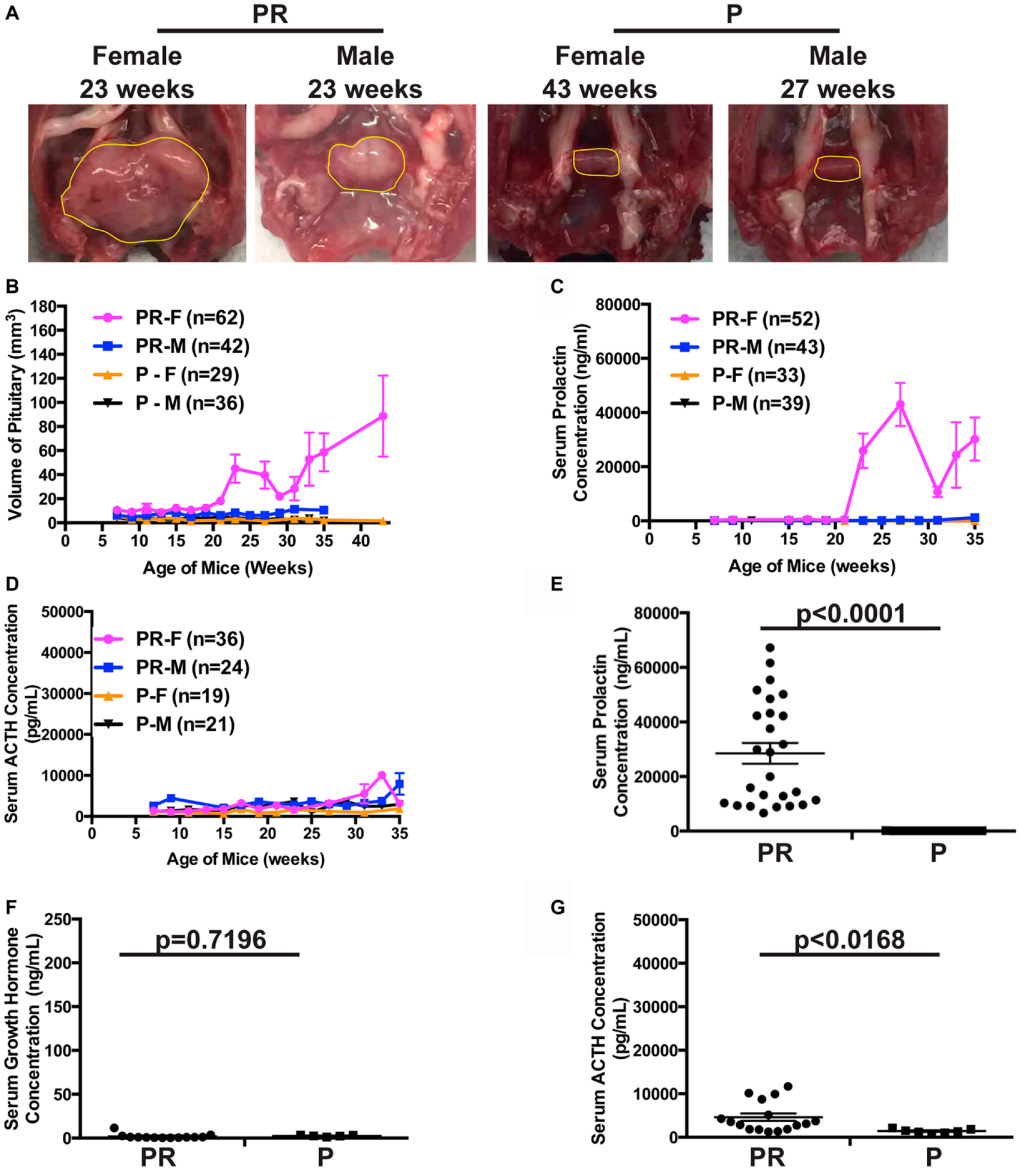
Deletion of TSG *Pten* alone led to PitNETs. (**A**) Gross pathology of pituitary in female and male PR and control P mice. Normal pituitary is cylindrical in shape as seen in control P mice. Pituitaries and PitNETs were circled in yellow lines inside the mouse skull. (**B**) Evaluation of the sizes of pituitaries in three-week interval starting at 6 weeks in female and male PR and control P mice at scheduled autopsy. (**C**) Serum prolactin levels measured by ELISA assay in female and male PR and control P mice as they age. (**D**) Serum ACTH levels measured by ELISA assay in female and male PR and P mice as they age. (**E**–**G**). Serum ELISA hormone assays in female PR mice with PitNETs and control female P mice of the same age with shown *p*-values. (E) prolactin, (F) growth hormone, and (G) ACTH.

Since it has been reported that PTEN may play a role in suppressing PitNETs in mice [48, 49], we evaluated whether the deletion of *Pten* alone led to PitNETs. We constructed *Pten^flox/flox^* RIP-Cre (PR) mice and monitored the growth of mice along with control *Pten^flox/flox^* (P) mice for up to forty-three weeks. All mice were viable and healthy. Autopsies of the brains of these mice every other week starting at seven weeks showed that pituitaries grew gradually in PR mice and eventually developed into tumors in female mice while a normal size pituitary was maintained in both female and male control P mice (Figure 5A and 5B). Gender bias was observed in pituitary growth and tumor development. Female mice displayed faster growth of pituitaries and developed earlier PitNETs. Consistent with this observation, significantly elevated serum prolactin levels were observed in female PR mice as PitNETs developed (Figure 5C), while serum GH levels were normal in both PR and P mice (data not shown) and serum ACTH levels were slightly increased in both male and female PR mice older than twenty-seven weeks compared to age- and sex-matched control P mice (Figure 5D). Female PR mice with PitNETs had dramatically elevated prolactin levels, normal GH levels, and slightly but significantly increased ACTH levels compared to age matched control female P mice (Figure 5E–5G), further confirmed by IHC staining (data not shown). These results indicated that PitNETs in female PR mice originated from anterior lobe while intermediate lobe was slightly enlarged as the pituitary grew in both female and male PR mice. The PitNETs were prolactinomas. Taken together, deletion of *Pten* alone led to PitNETs in mice, and PTEN plays a role in anterior and intermediate lobes of pituitary.

### *Pten* plays a more significant role than Men1 in suppressing PitNETs

**Figure 6:**
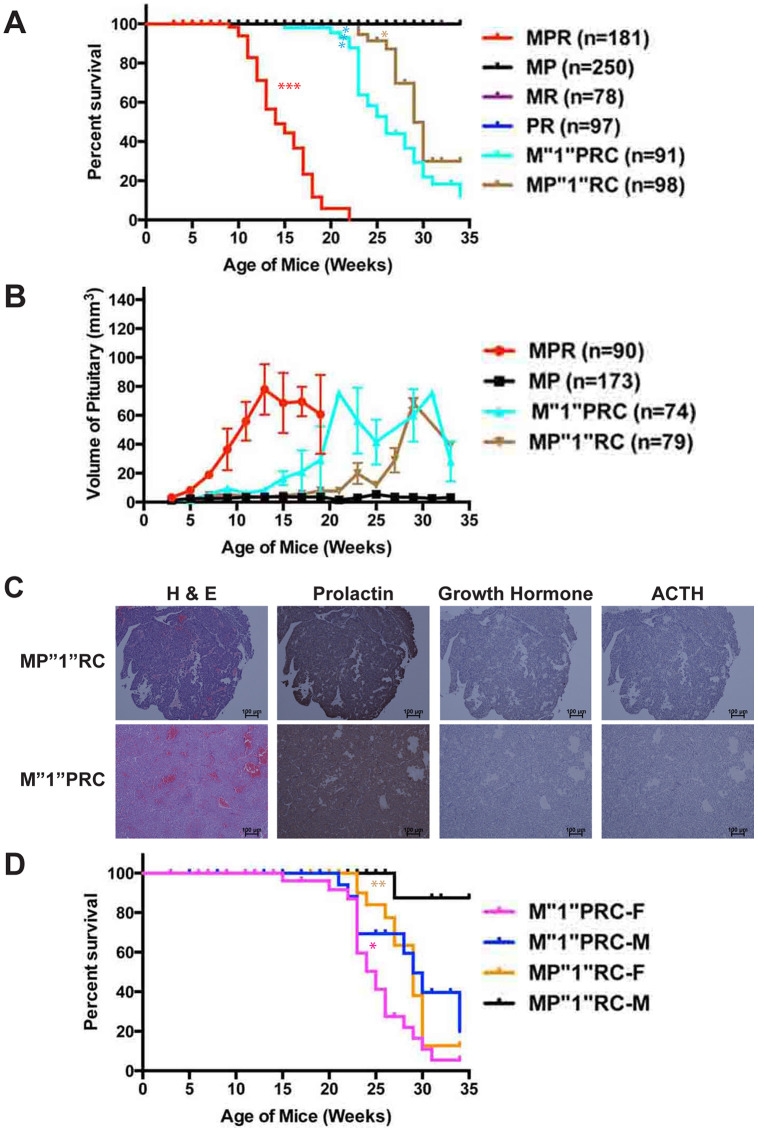
*Pten* plays a more significant role in suppressing PitNETs compared to *Men1*. (**A**) Survival curve of M”1”PRC and MP”1”RC mice in comparison with MPR, MR, PR, and MP mice. M”1”PRC mice showed significantly longer life span than MPR mice (*p* < 0.0001), but shorter life span than MP”1”RC mice (*p* < 0.0039) and PR mice (*p* < 0.0001). MP”1”RC mice showed shorter lifer span than MR mice (*p* < 0.035). (**B**) Evaluation of the pituitary sizes of M”1”PRC and MP”1”RC mice in comparison with MPR and MP mice as they age. (**C**) H & E, and IHC staining of prolactin, growth hormone and ACTH showed that both M”1”PRC and MP”1”RC mice developed prolactinomas. (**D**) Gender bias in survival was observed in M”1”PRC and MP”1”RC mice. Female M”1”PRC (*p* < 0.0305) and MP”1”RC (*p* < 0.0025) mice showed shorter life sans than corresponding male mice. ^***^ *p* < 0.0001, ^**^ *p* < 0.01, ^*^ *p* < 0.05.

Since PR mice developed PitNETs earlier and faster than MR mice (Figures 1D and 5B), we investigated whether *Pten* plays a more important role in pituitary tumorigenesis than *Men1* using the same strategy as above for *Rb1*. We performed survival, pathological and histological analyses of mice with these genotypes: *Men1^flox/+^ Pten^flox/flox^* RIP-Cre (M”1”PRC), *Men1^flox/flox^ Pten^flox/+^* RIP-Cre (MP”1”RC) and *Men1^flox/+^ Pten^flox/+^* RIP-Cre (M”1”P”1”RC) up to thirty-four weeks. These mice were the littermates of MPR mice described in [48]. Double heterozygous M”1”P”1”RC mice were viable and healthy during the study period. The Kaplan–Meier survival curve demonstrated that M”1”PRC mice had shorter life spans than MP”1”RC mice (*p* < 0.0039) (Figure 6A). The median survival of M”1”PRC mice was twenty-six weeks and that of MP”1”RC mice was twenty-nine weeks during the study period. MP”1”RC mice (*p* < 0.0353) and M”1”PRC mice (*p* < 0.0001) had decreased survival compared to MR and PR mice, respectively, but longer life spans than MPR mice (*p* < 0.0001). Evaluation of the growth pace of the pituitaries in the cohort of mice in two-week intervals was consistent with the pattern of the survival curve suggesting that faster development and larger PitNETs resulted in shorter life spans (Figure 6B), indicating these mice were sick due to development of PitNETs. Besides this, pituitaries grew faster in MPR mice than M”1”PRC and MP”1”RC mice; faster in M”1”PRC mice than PR mice and faster in MP”1”RC mice than MR mice (Figures 6B, 5B, and 1D), indicating that the suppression of PitNETs by PTEN and Menin was dosage-dependent and deletion of *Pten* had a stronger effect on pituitary tumorigenesis than deletion of *Men1*.

Consistent with MR and PR mice developing prolactinomas, both M”1”PRC (*n* = 10) and MP”1”RC (*n* = 10) mice developed prolactinomas (Figure 6C) based on IHC staining of prolactin, GH, and ACTH. Consistent with prolactinomas development in these mice, a gender bias in survival was observed (Figure 6D). Female M”1”PRC (*p* < 0.0305) and MP”1”RC (*p* < 0.0025) mice had significantly shorter life spans than corresponding male mice. Collectively, PTEN plays a more important role in suppressing PitNETs than Menin.

### *Rb1* and *Pten* function cooperatively to accelerate PitNETs and death

**Figure 7:**
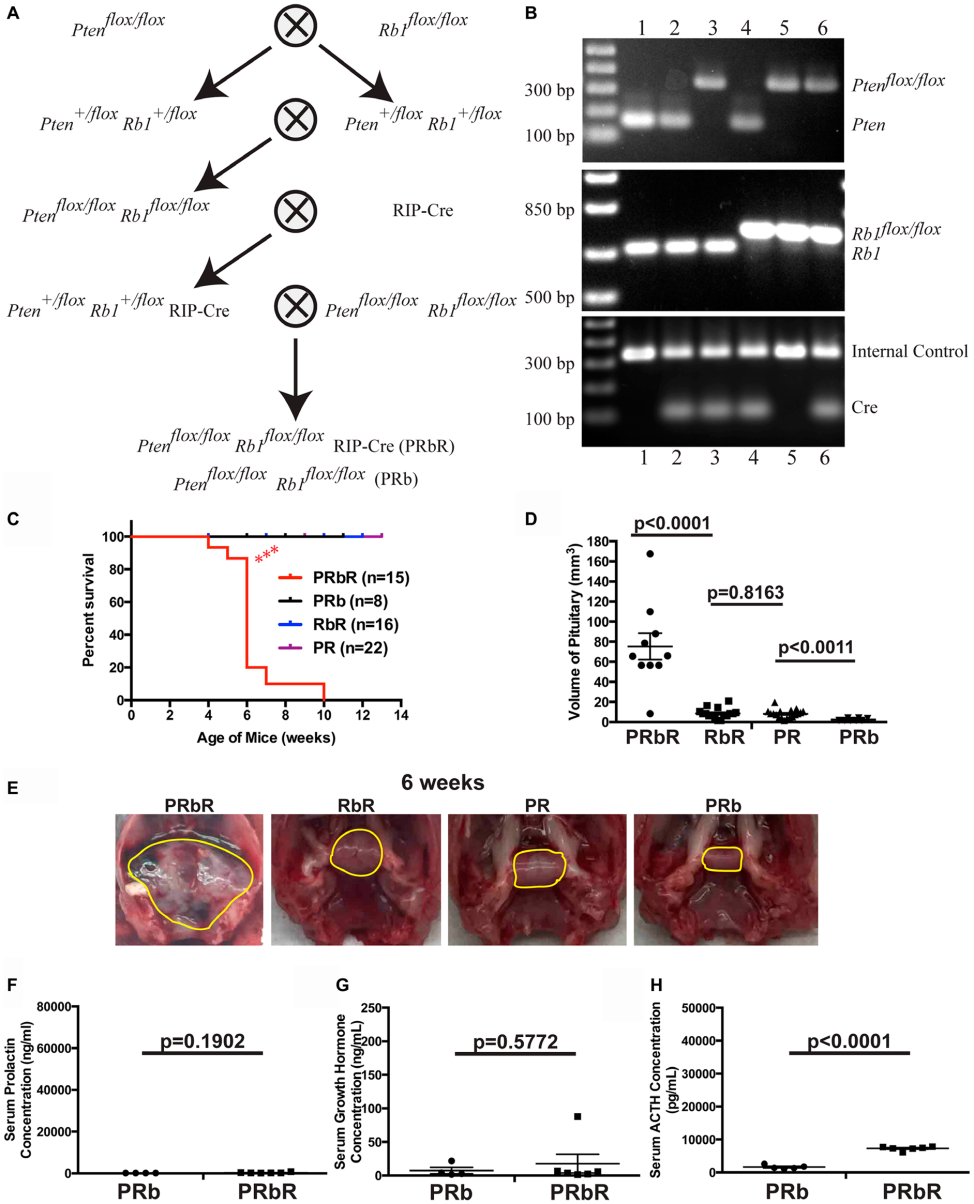
*Pten* and *Rb1* function cooperatively to suppress PitNETs. (**A**) Diagram of the strategy used to generate compound PRbR and PRb mice. (**B**) Representative genotyping results of the litters in A by PCR analysis using tail genomic DNA. Genotypes of each lane: 1-WT, 2-RIP-Cre, 3-*Pten^flox/flox^* RIP-Cre (PR), 4-*Rb1^flox/flox^* RIP-Cre (RbR), 5-*Pten^flox/flox^ Rb1^flox/flox^* (PRb), 6-*Pten^flox/flox^ Rb1^flox/flox^* RIP-Cre (PRbR). (**C**) Survival curves showed that concomitant loss of *Rb1* and *Pten* accelerated death in PRbR mice. PRbR mice showed significantly shorter lifer span (*p* < 0.0001) than PR, RbR, and PRb mice. (**D**) The sizes of PitNETs in double deletions PRbR mice at death were significantly larger than that in single deletion RbR, PR, and wild-type control PRb mice of the same age as shown *p*-values. (**E**) Gross pathology of pituitary in double deletions PRbR, single deletion RbR and PR, and wild-type control PRb mice at 6 weeks. A normal pituitary is cylindrical in shape as seen in wild-type control PRb mice. Pituitaries or PitNETs were circled in yellow lines inside the mouse skull. (**F**–**H**) Serum ELISA hormone assays in PRbR and PRb mice with shown *p*-values. (F) prolactin, (G) growth hormone, and (H) ACTH.

If both PTEN and pRB play important roles in suppressing PitNETs, we predicted that mice with double deletion of *Pten* and *Rb1* would exhibit cumulative effects on pituitary tumorigenesis. We constructed tissue-specific double homozygous deletions of *Pten* and *Rb1* in pancreas and pituitary using the same strategy as used for MRbR mice (Figure 7A), confirmed the correct genotypes of the compound mice by PCR analysis using tail DNA (Figure 7B), and monitored the growth of these double homozygous deletions *Pten^flox/flox^ Rb1^flox/flox^* RIP-Cre (PRbR) and wild-type control *Pten^flox/flox^ Rb1^flox/flox^* (PRb) mice. Consistent with our discovery of more important roles for *Rb1* and *Pten* than *Men1* in pituitary, PRbR (*n* = 14) mice showed symptoms of PitNETs such as loss of vision, tilted head/body, and circular gait path starting at four weeks and did not live beyond ten weeks (Figure 7C), indicating that PitNETs developed much earlier in PRbR mice than in single deletion RbR and PR mice and double deletions MRbR and MPR mice [48]. Wild-type control PRb mice and single deletion PR and RbR mice were viable and healthy up to twelve weeks.

Autopsies of the brains showed that PRbR mice had large PitNETs, with sizes at death that were almost the same as the PitNETs of MRbR and MPR at death (Figures 7D, 7E, 4A, and reference 48). Pancreas in these sick mice was normal macroscopically and histologically. Single deletion PR and RbR mice of the same age only had slightly enlarged pituitaries not significantly different from each other, but both significantly larger than wild-type control PRb mice (Figure 7D and 7E). Serum ELISA hormone assays indicated that PRbR mice had significantly higher serum ACTH concentrations than PRb mice, while serum prolactin and GH concentrations showed no significant difference from PRb mice (Figure 7F–7H). IHC staining of the PitNETs from sick PRbR (*n* = 8) mice confirmed ELISA assay results (data not shown). Thus, these pituitary tumors in PRbR mice were ACTH-secreting PitNETs. Collectively, our data indicated that deletion of *Rb1* had a cooperative tumorigenesis phenotype with the deletion of *Pten* in pituitary. Both pRB and PTEN play important roles in suppressing PitNETs.

### *Trp53* and *Pten* had weak cooperative function in suppressing pituitary growth

It has been reported that heterozygous *Rb1* deletion and homozygous *Trp53* deletion has cooperative effects on neuroendocrine tumorigenesis while heterozygous *Men1* deletion and homozygous *Trp53* deletion has non-synergistic effects on tumorigenesis in mice [31, 54]. We asked the question whether *Trp53* has cooperative effects with *Pten* in suppressing NETs. We constructed tissue-specific double homozygous deletions of *Trp53* and *Pten* in pancreas and pituitary (Supplementary Figure 1A) to investigate whether *Trp53* and *Pten* have a cooperative role in NETs. The genotypes of the compound mice were confirmed by PCR analysis using tail genomic DNA (Supplementary Figure 1B). We also constructed double homozygous deletions of *Trp53* with *Rb1* and *Men1*, respectively. Confirmation of the representative genotypes was shown in Supplementary Figure 1C and 1D.

**Figure 8:**
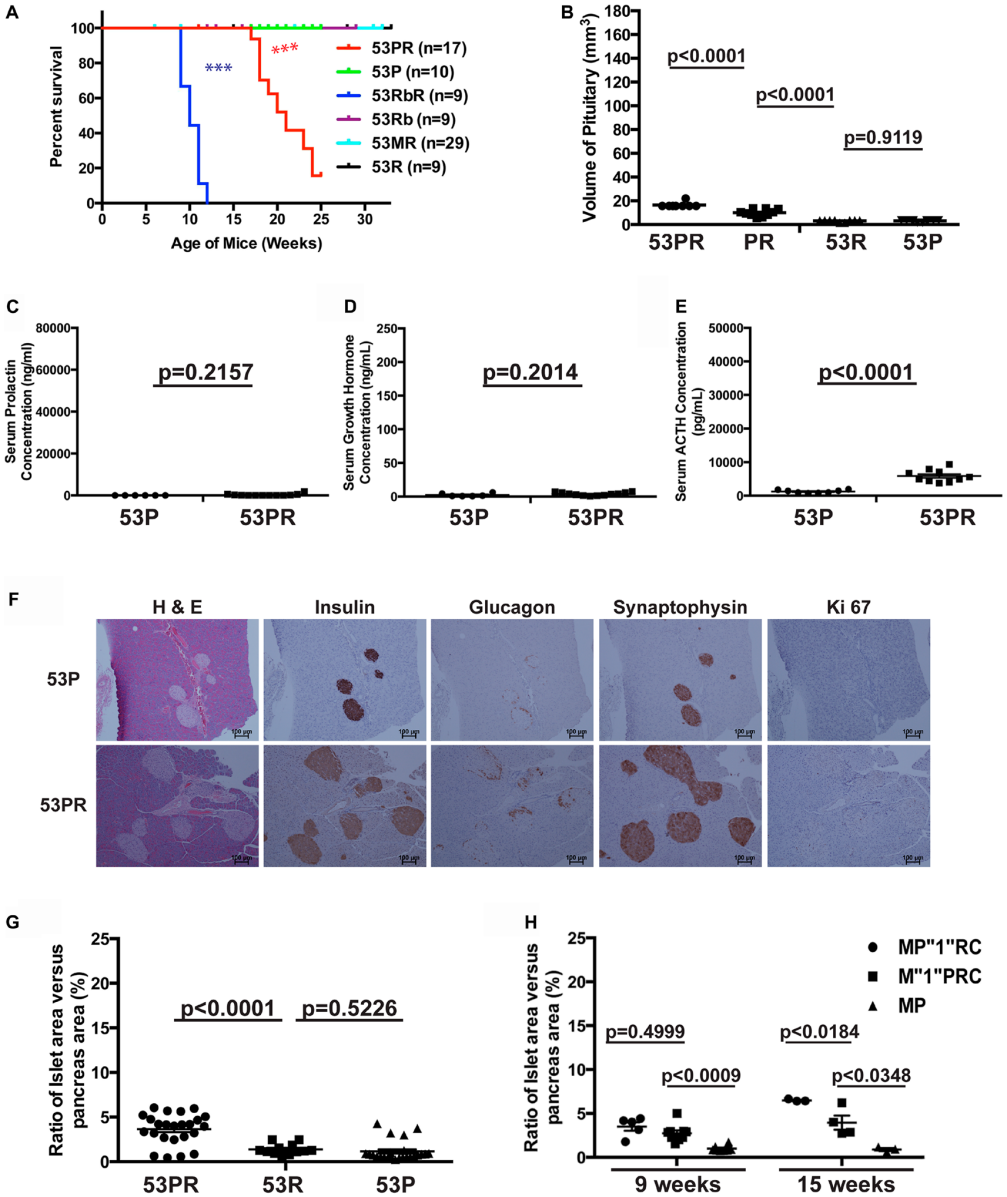
*Trp53* and *Pten* had weak cooperative function in suppressing pituitary growth. (**A**) Survival curve of 53PR and control 53P mice, as well as 53RbR, 53MR and their corresponding control mice. The end point for 53PR mice was the paralysis of hind limbs in our study. 53RbR mice had significantly shorter life spans (*p* < 0.0001) than 53 PR mice, which has significantly shorter life spans (*p* < 0.0001) than 53 MR and wild-type control mice. (**B**) The sizes of pituitaries in 53PR mice at the study endpoint were larger than that in single deletion PR and 53R mice and wild-type control 53P mice matched by age and sex as shown *p*-values. (**C**–**E**) Serum ELISA hormone assays in 53PR and 53P mice with shown *p*-values. (C) prolactin (D) growth hormone (E) ACTH. (**F**) H & E, IHC staining of Insulin, Glucagon, Synaptophysin, and Ki67 of pancreas sections from 53P and 53PR mice. (**G**) Quantitative comparison of the ratio of the islets area per pancreas area between 53PR, 53R and 53P mice at 17 weeks with shown *p*-values. (**H**) Quantitative comparison of the ratio of the islets area per pancreas area among M”1”PRC, MP”1”RC, and wild-type control MP mice at 9 and 15 weeks with shown *p*-values. ^***^ *p* < 0.0001.

We monitored the growth of compound mice with double deletions *Trp53^flox/flox^ Pten^flox/flox^* RIP-Cre (53PR) and wild-type control *Trp53^flox/flox^ Pten^flox/flox^* (53P) without RIP-Cre transgene, as well as compound mice with double deletions *Trp53^flox/flox^ Rb1^flox/flox^* RIP-Cre (53RbR) and *Trp53^flox/flox^ Men1^flox/flox^* RIP-Cre (53MR), single deletion *Trp53^flox/flox^* RIP-Cre (53R), and corresponding wild-type controls *Trp53^flox/flox^ Rb1^flox/flox^* (53Rb), *Trp53^flox/flox^ Men1^flox/flox^* (53M) without RIP-Cre transgene. 53PR mice showed paralysis of hind limbs starting at around seventeen weeks but did not show symptoms of PitNETs. The end point of 53PR mice in this study was the paralysis of hind limbs according to IACUC while control 53P mice of the same age and sex were viable and healthy (Figure 8A). Autopsies of the brains showed that the sick 53PR mice had intact cylindrical, but slightly and significantly enlarged pituitaries (Figure 8B). Histology of the pituitaries showed normal pituitary staining with enlarged intermediate lobe. Serum ELISA hormone assays confirmed the slightly but significantly increased ACTH levels in 53PR mice compared to control 53P mice while prolactin and GH levels were similar (Figure 8C–8E), implying that the intermediate lobe of the pituitaries grew slightly bigger in 53PR mice than control 53P mice.

Similar to what has been reported on mice with homozygous *Trp53* and heterozygous *Men1* or *Rb1* [31, 54], double deletions 53MR mice were viable and healthy up to thirty-two weeks. Double deletions 53RbR mice showed symptoms of PitNETs such as loss of vision, tilted head/body, circular gait path starting at nine weeks and did not live beyond twelve weeks, while wild-type control mice 53Rb, and single deletion mice 53R and RbR of the same age and sex were viable and healthy (Figures 8A and 1C). Large PitNETs were observed in sick 53RbR mice while normal or slightly enlarged pituitaries were found in wild-type control 53Rb or single deletion RbR and 53R mice of the same age (Supplementary Figure 2A). Pituitaries from single deletion 53R mice were similar to those from wild-type control 53Rb mice of the same age, consistent with what has been reported that deletion of *Trp53* did not lead to PitNETs in mice. IHC staining of the PitNETs from 53RbR mice displayed two types of cells: ACTH-secreting tumors (Supplementary Figure 2B, middle panel) and transformed cells with heterogeneous prolactin, GH and ACTH staining (Supplementary Figure 2B, bottom panel). Serum ELISA assays confirmed significantly high ACTH levels in 53RbR mice (data not shown). Thus, pRB has cooperative function with TRP53 in suppressing PitNETs, consistent with the reported cooperative function between the two TSGs [31] and pRB plays a more important role in pituitary tumorigenesis than TRP53.

Since the sizes of the pituitaries at the study endpoint (17–25 weeks) in 53PR mice were slightly but significantly larger than that in single deletion PR and 53R mice or wild-type control 53P mice of the same age but much smaller than that in PRbR, 53RbR and MPR mice at death at younger ages (Figures 8B, 7D, Supplementary Figure 2A, and reference 48), TRP53 and PTEN had weak cooperative function in suppressing pituitary growth in mice.

Further examination of pancreas in the sick 53PR mice showed normal pancreas with increased numbers of small round islets and normal hormone distribution (Figure 8F). Quantitative analysis of the ratio of the islets area per pancreas area indicated that double deletions 53PR mice have significantly increased islets area ratio compared to that in the single deletion 53R and wild-type control 53P mice (Figure 8G), but have no significant difference compared to that in the single deletion PR mice (*p* = 0.9689) (Figure 3D), indicating that the increased numbers of islets in double deletions 53PR mice were due to the effect from deletion of *Pten* alone. Thus, TRP53 and PTEN have no cooperative function in islet lesions at the study end point.

Examination of pancreas in 53MR mice did not show any synthetic islet lesions compared to MR mice alone, which is consistent with what has been reported [54]. Macroscopic examination of the pancreas in 53RbR mice displayed multifocal bloody tumors starting at nine weeks. IHC staining of pancreas displayed large tumors with abnormal hormone distribution of a cells - negative staining of glucagon while wild-type control 53Rb mice showed small and round islets with normal peripheral α cell distribution and single deletion 53R (data not shown) and RbR mice of the same age displayed normal and hyperplastic islets (Supplementary Figures 2C and 3B). Immunoreactivity for insulin, neuroendocrine markers chromogranin A and synaptophysin (data not shown) indicated these were PanNETs. Different from WD G1/G2 PanNETs developed in MRbR mice, 53RbR mice developed G3 PanNETs with average Ki67 index of 29.7% (*n* = 4). Taken together, our data showed that TRP53 did not function cooperatively with PTEN in islet lesions while TRP53 function cooperatively with pRB to suppress PanNETs and had no cooperative function with MEN1 in islet lesions as reported [31, 54]. Thus, TRP53 is dispensable with the intact pRB, but it seems that the role of TRP53 is indispensible in suppressing PitNETs and PanNETs in the absence of pRB.

The paralysis of limbs in 53PR mice may be due to the leaky expression of RIP-Cre transgene in nerves or muscles, which led to double deletions of *Pten* and *Trp53*. We observed paralysis of hind limbs in MPR mice that lived longer than seventeen weeks. We did not observe the paralysis in single deletion mice PR, MR or 53R up to forty-three weeks or longer. The paralysis was due to the cooperative function between the tumor suppressors TRP53 and PTEN. Since this is out of the scope of this paper, we did not pursue further what caused the paralysis of hind limbs in 53PR mice.

## DISCUSSION

**Figure 9:**
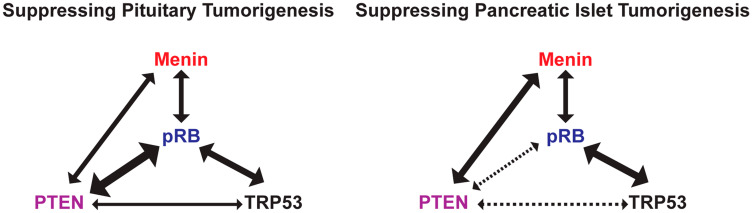
Genetic interactions between TSGs in suppressing pituitary and pancreatic islet tumorigenesis. Thick solid lines with double arrows meant the strong cooperative interaction; thin solid lines with double arrows meant the weak cooperative interaction; dotted lines with double arrows meant that cooperative interactions were not be able to be determined in this study; no lines meant no cooperative interaction.

It is well known that most human cancers result from the accumulation of multiple genetic changes, including activating mutations in oncogenes and the loss of function mutations in TSGs [55]. To understand the molecular mechanism of pituitary and pancreatic islet tumorigenesis, this paper and our recently published paper [48] together present a systematic evaluation of genetic interactions between the tissue-specific loss of common TSGs—*Rb1*, *Trp53*, *Pten*, and *Men1*—in mice. TSGs were deleted in pituitary and pancreatic islets using the Cre-LoxP system with Cre under the control of the rat insulin II promoter. Our systematic pairwise homozygous deletions of the TSGs directly illustrated the genetic interactions of these TSGs in suppressing PitNETs and PanNETs (Figure 9). Our data demonstrated that pRB had the strongest cooperative function with PTEN in suppressing PitNETs. pRB had strong cooperative function with TRP53 and Menin, respectively, in suppressing PitNETs and PanNETs. TRP53 had weak cooperative function with PTEN in suppressing pituitary growth. We also demonstrated that deletion of *Pten* singly led to prolactinomas in female mice and slow growth of the intermediate lobe of pituitaries in both female and male mice. Deletion of *Rb1* alone led to islet hyperplasia in pancreas and *Trp53* may play a role mainly in the intermediate lobe of the pituitary.

The genetic analysis of the tumorigenic phenotypes in single and double deletions of TSGs in pituitary implied that the order of functional importance of TSGs from most to least in pituitary tumorigenesis was *Rb1*, *Pten*, *Men1*, and *Trp53*. Mice with single deletion of *Rb1* using the RIP-Cre system developed fully penetrant ACTH-secreting PitNETs in our study, which is consistent with previous reports on heterozygous *Rb1^+/–^* mice [56]. Mice with single deletion of *Pten* in this study and *Men1* [37] developed prolactinomas, mainly in female mice at a latency. Mice with single deletion of *Trp53* did not develop PitNETs. These results indicate that deletion of *Rb1* had the strongest effect on the pituitary tumorigenesis.

M”1”RbRC mice developed PitNETs faster and more severely than MRb”1”RC mice further supporting a more important role for *Rb1* than *Men1*. Survival, pathology and phenotype of M”1”PRC and MP”1”RC mice showed that M”1”PRC mice developed PitNETs faster than MP”1”RC mice, indicating that deletion of *Pten* had stronger effect on the development of PitNETs than deletion of *Men1*, and *Pten* played a more important role in pituitary tumorigenesis than *Men1*. Consistent with the important roles of *Rb1* and *Pten* in pituitary tumorigenesis, PRbR mice developed fully penetrant PitNETs the earliest at around six weeks of age. Although deletion of *Trp53* itself did not lead to PitNETs, double deletions with *Rb1* led to fully penetrant ACTH-secreting PitNETs by nine weeks, and double deletions with *Pten* led to slowly growing pituitaries with high serum ACTH concentrations, suggesting that *Trp53* played a role in pituitary tumorigenesis, perhaps mainly in the intermediate lobe of the pituitary. Given the fundamental difference in murine versus humans where ACTH corticotrophs are largely found in the anterior pituitary in humans and in the intermediate lobe in mice, it might be expected that *p53* mutations in humans would associate with anterior pituitary tumors.

Based on the age of onset and the rate of growth of tumors, we propose that deletion of *Rb1* affects the initiation and progression of pituitary tumorigenesis, deletion of *Pten* or of *Men1* has more of an effect on initiation than progression of pituitary tumorigenesis, while deletion of *Trp53* influences progression rather than initiation of pituitary tumorigenesis. Further characterization of multistep tumorigenesis and complex molecular signatures involved in pituitary tumorigenesis could be investigated through global transcriptional profiling of the PitNETs from these mice. Dissection of the molecular signatures involved in the pituitary tumorigenesis will help unravel molecular mechanisms. Collectively, pRB and PTEN/PI3K/AKT pathways play important roles in suppressing PitNETs in mice.

It is widely known that *MEN1* plays an important role in human PanNETs than other TSGs *RB1*, *TP53*, and *PTEN* based on genetic and genomic analysis of the human PanNETs. Here we performed targeted deletions of these four TSGs singly and pairwise in pancreatic islets in mice and demonstrated directly that deletion of *Men1* had the strongest effect on islet tumorigenesis, consistent with what is widely accepted in man. At fifteen weeks, MR mice did not show significant difference in the ratio of islets area per pancreas area from PR mice, but both MR and PR mice showed significantly larger islets area per pancreas area compared with RbR mice (Figure 3D), suggesting that deletion *Men1* or *Pten* had stronger effects on islet hyperplasia than deletion of *Rb1*. MR mice developed PanNETs after twenty-three weeks and with high frequency and severity increasing after thirty-five weeks (our study and [37]). Evaluation of islets area per pancreas area in M”1”PRC and MP”1”RC mice showed that MP”1”RC mice had significantly larger islets area per pancreas area compared to M”1”PRC mice at fifteen weeks (Figure 8H), supporting a more important role for Menin in islet tumorigenesis than PTEN.

Double homozygous deletions of *Men1* and *Pten* accelerated fully penetrant PanNETs [48] while double homozygous deletions of *Men1* and *Rb1* accelerated development of PanNETs in around 50% of mice, supporting a more important role for PTEN in islet tumorigenesis than pRb. Two possibilities could explain the function of pRb in islet lesions. One is that pRb and Menin may have some overlapping function in suppressing PanNETs. The other is that pRb has limited function in islet lesions. Due to PitNET development and death in RbR and MRbR mice, pancreas could not be examined at later time points. Using a different promoter with our experimental system would help address this question. Deletion of *Rb1* alone and in combination with *Men1* in mice containing the Cre transgene driven by the mouse insulin 1 promoter (MIP-Cre) [48] would lead to deletion of TSGs in pancreatic islets only. This would help us examine whether *Rb1* plays a role in islet tumorigenesis and whether *Men1* and *Rb1* have any overlapping function in islet tumorigenesis.

In this RIP-Cre system, pancreas sections from PRbR and 53PR mice were not evaluated at later time points due to early death in PRbR mice and hind limb paralysis in 53PR mice. Mice with double homozygous deletions of TSGs in pancreatic islets only using MIP-Cre system would help us understand whether double deficiencies of *Pten* with *Rb1* or *Trp53* have any cooperative tumorigenic effects on pancreatic islets. Double homozygous deletions of *Rb1* and *Men1* developed WD G1/G2 PanNETs while double homozygous deletions of *Rb1* and *Trp53* developed WD G3 PanNETs and double homozygous deletions of *Men1* and *Trp53* did not show a synergistic effect on islet tumorigenesis, as reported earlier [54]. These results indicate that even if Menin and pRb or Menin and TRP53 has overlapping functions in suppressing PanNETs, they also have mutually exclusive and independent functions in pancreatic islets.

Taken together, the order of functional importance of TSGs in islet tumorigenesis from most to least important is *Men1*, *Pten*, *Rb1*, and *Trp53*. Based on the age of onset, frequency and severity of tumorigenesis, we propose that deletion of *Men1* may affect more on initiation than progression of islet tumorigenesis, deletion of *Rb1* or of *Pten* may affect initiation of islet tumorigenesis, while deletion of *Trp53* may affect the progression of islet tumorigenesis. Menin and PTEN/PI3K/AKT pathways play important roles in suppressing PanNETs in mice.

In summary, our data clearly demonstrate that TSGs *Rb1*, *Pten*, *Men1*, and *Trp53* have distinct tissue specificity in neuroendocrine tumorigenesis in mouse and likely in man. The mouse models here and deletion of these TSGs in MIP-Cre mice will help further our understanding the molecular function of these TSGs and their pathways in PitNET and PanNET pathogenesis, which will help develop targeted novel therapeutic options in treating human patients.

## MATERIALS AND METHODS

### Genetic crosses and molecular analysis

To generate compound mice *Men1^flox/flox^ Rb1^flox/flox^* RIP-Cre (MRbR) (Figure 1A), *Men1^flox/flox^* mice (129S (FVB)-*Men1^tm1.2Ctre^*/J, stock number 005109, The Jackson Laboratory, USA) were first crossed with *Rb1^flox/flox^* mice (FVB;129-*Rb1^tm2Brn^*/Nci, stock number: 01XC1, The Frederick National Laboratory for Cancer Research, USA) to generate heterozygous *Men1^+/flox^ Rb1^+/flox^* mice. The resulting mice were then intercrossed to generate *Men1^flox/flox^ Rb1^flox/flox^* (MRb) mice. The resulting double homozygous floxed mice were then crossed with RIP-Cre mice (C57BL/6-Tg (Ins2-cre) 25Mgn/J, stock number: 003573, The Jackson Laboratory, USA) to generate *Men1^+/flox^ Rb1^+/flox^* RIP-Cre mice. These mice were further crossed back to MRb mice to generate the desired double homozygous deletions MRbR compound mice, and corresponding littermates MRb, *Men1^flox/+^ Rb1^flox/flox^* RIP-Cre (M”1”RbRC), *Men1^flox/flox^ Rb1^flox/+^* RIP-Cre (MRb”1”RC), *Men1^flox/+^ Rb1^flox/+^* RIP-Cre (M”1”Rb”1”RC). Confirmation of the genotypes in mice was evaluated by PCR using tail genomic DNA (Figure 1B). Tissue-specific expression of RIP-Cre in pancreatic islets and brain was confirmed previously [48]. Due to PitNETs developed in MRbR mice, MRbR mice were infertile. All of the MRbR mice and their littermates were generated through the series of crosses. Compound mice *Pten^flox/flox^ Rb1^flox/flox^* RIP-Cre (PRbR), *Trp53^flox/flox^ Pten^flox/flox^* RIP-Cre (53PR) and *Trp53^flox/flox^ Rb1^flox/flox^* RIP-Cre (53RbR) were generated using the same strategy as for MRbR mice, except that *Pten^flox/flox^* mice (C;129S4-*Pten^tm1Hwu^*/J, stock number: 004597, The Jackson Laboratory, USA) or/and *Trp53^flox/flox^* mice (B6.129P2-*Trp53^tm1Brn^*/J, stock number: 008462, The Jackson Laboratory, USA) were used (Figure 7A and Supplementary Figure 1A). Confirmation of the genotypes in mice was evaluated by PCR using tail genomic DNA (Figure 7B and Supplementary Figure 1B and 1C). All of the PRbR, 53PR, 53RbR mice and their littermates were generated through the series of crosses due to sickness of these mice. The first generation of *Trp53^flox/flox^ Men1^flox/flox^* RIP-Cre (53MR) mice was generated using the same strategy. Then 53 MR mice were crossed with each other to generate cohorts of 53MR mice and confirmation of the genotypes was evaluated by PCR using tail DNA (Supplementary Figure 1D). *Men1^flox/+^ Pten^flox/flox^* RIP-Cre (M”1”PRC), *Men1^flox/flox^ Pten^flox/+^* RIP-Cre (MP”1”RC) and *Men1^flox/+^ Pten^flox/+^* RIP-Cre (M”1”P”1”RC) mice were the littermates from the crosses to generate *Men1^flox/flox^ Pten^flox/flox^* RIP-Cre (MPR) mice [48]. Single deletion *Rb1^flox/flox^* RIP-Cre (RbR) mice were produced by generating heterozygous *Rb1^+/flox^* RIP-Cre animals through the first cross of *Rb1^flox/flox^* mice with RIP-Cre mice and then crossing the resulting *Rb1^+/flox^* RIP-Cre mice with *Rb1^flox/flox^* mice. Single deletion *Trp53^flox/flox^* RIP-Cre (53R), *Pten^flox/flox^* RIP-Cre (PR) and *Men1^flox/flox^* RIP-Cre (MR) mice were produced through a strategy similar to that used to produce RbR mice. All cohorts were in a mixed genetic background. Animals were housed in a temperature-, humidity-, and light-controlled room (12-hour light/dark cycle) and with free access to food and water. All animal experiments were conducted according to the research guidelines set forth by the Institutional Animal Care and Use Committee (IACUC) of Rutgers, the State University of New Jersey, USA.

Animals were genotyped by standard genomic PCR techniques using tail DNA. Tail Genomic DNA was isolated using Promega Nuclei Lysis solution/EDTA (Promega Corporation, USA). Primers for PCR analysis were ordered from Integrated DNA Technologies (IDT) based on Vendors’ recommendations [57] (Table 4). PCR fragments from tail genomic DNA were amplified using a thermal cycler (Veriti, the applied biosystems, USA) (94°C, 3 min; 94°C, 30 sec, 60°C, 1 min, 72°C, 1 min, for 40 cycles; 72°C, 7 min, or as Vendor’s recommendations).

**Table 4:** List of primer sequences for genotyping

Gene Name	Primer Name	Primer sequences
**Cre**	oIMR1084	5′-GCG GTC TGG CAG TAA AAA CTA TC-3′
	oIMR1085	5′-GTG AAA CAG CAT TGC TGT CAC TT-3′
	oIMR7338	5′-CTA GGC CAC AGA ATT GAA AGA TCT-3′
	oIMR7339	5′-GTA GGT GGA AAT TCT AGC ATC ATC C-3′
***Men1***	oIMR1484	5′-CCC ACA TCC AGT CCC TCT TCA GCT-3′
	oIMR1485	5′-CCC TCT GGC TAT TCA ATG GCA GGG-3′
	primer C-R	5′-CGG AGA AAG AGG TAA TGA AAT GGC-3′
***Rb1***	R007	5′-GGC GTG TGC CAT CAA TG-3′
	R008	5′-AAC TCA AGG GAG ACC TG-3′
***Pten***	oIMR9554	5′-CAA GCA CTC TGC GAA CTG AG-3′
	oIMR9555	5′-AAG TTT TTG AAG GCA AGA TGC-3′
***Trp53***	oIMR8543	5′-GGT TAA ACC CAG CTT GAC CA-3′
	oIMR8544	5′-GGA GGC AGA GAC AGT TGG AG-3′

### Macroscopic and microscopic evaluation of pituitary and pancreas

This was basically performed the same as what described in [48]. To evaluate pituitary size inside the brain skull macroscopically, a ruler was used to measure the length, width and height of the pituitary at autopsy. The volume of a pituitary was calculated with the formula V=(p/6) × (length × width × height). To evaluate pituitary histology, the brain skull with intact pituitary was fixed in 10% buffered formalin solution (Fisher Scientific, Inc., USA) for 48 h at 4°C. After fixation, pituitary was removed from the brain skull gently and wrapped inside a biopsy paper, then washed in 50% ethanol and transferred to 70% ethanol for paraffin embedding, further sectioned and stained in the immunohistochemistry experiments as described previously. Pancreas was examined from head to tail if there are any nodules/tumors macroscopically. To score PanNETs microscopically, three or four pancreas sections were sectioned 120 μm-apart from each mouse and stained with hematoxylin and eosin (H & E), insulin and glucagon. One or more islets of ≥ 1 mm in diameter in any of the three or four sections with positive insulin staining were scored as tumor for that mouse based on histologic examination. To evaluate islet lesions quantitatively, the insulin-stained pancreas sections from the three or four sections taken 120 μm-apart per mouse were digitized at 20 ×. The ratio of islets area (insulin positive area) per pancreas area for each mouse was calculated and graphed as described in [48]. For quantification of IHC positive staining for Ki-67, the areas with the highest density of Ki-67 reactivity among tumor cells were first identified. At least 1000 cells were counted at 20× magnification in these high Ki-67 density areas in a minimum of three mice. Antibodies used for immunohistochemistry experiments were the same as described in [48].

### Serum assays

All mice were fasted for 3–5 hours in the morning before blood collection. Blood glucose was measured with the ONE TOUCH Ultra2 blood glucose meter (Lifescan, Inc., USA). Serum insulin levels were measured with the Ultra Sensitive Mouse Insulin ELISA kit (Crystal Chem Inc., 90080, USA). Serum prolactin, growth hormone and ACTH levels were determined with the kits from Calbiotech (PR063F-100, USA), Millipore (EZRMGH-45K, USA) and Abcam Inc. (ab263880, USA), respectively. All experiments were performed based on manufacturers’ instructions and repeated at least twice independently.

### Statistical analysis

All statistical analyses and graphs were performed using GraphPad Prism version 6.0b software. The statistical significance of survival curves between two groups was analyzed using the log-rank (Mantel–Cox) test, and the statistical significance of serum hormone levels, of the ratio of islet area per pancreas area, of volume of pituitaries at death between two groups was analyzed using the unpaired *t*-test with Welch’s correction. *p* < 0.05 was considered significant.

## SUPPLEMENTARY MATERIALS



## Abbreviations

TSGs: Tumor suppressor genes
*MEN1* or *Men1*: Multiple endocrine neoplasia type 1
PTEN or *Pten*: Phosphatase and tensin homolog
pRB or *Rb1*: retinoblastoma susceptibility gene 1
NETs: neuroendocrine tumors
PitNETs: pituitary neuroendocrine tumors
PanNETs: pancreatic neuroendocrine tumors
GH: growth hormone
ACTH: adrenocorticotropin hormone
MRbR: *Men1^flox/flox^ Rb1^flox/flox^* RIP-Cre
MRb: *Men1^flox/flox^ Rb1^flox/flox^*
MR: *Men1^flox/flox^* RIP-Cre
RbR: *Rb1^flox/flox^* RIP-Cre
M”1”RbRC: *Men1^flox/+^ Rb1^flox/flox^* RIP-Cre
MRb”1”RC: *Men1^flox/flox^ Rb1^flox/+^* RIP-Cre
M”1”Rb”1”RC: *Men1^flox/+^ Rb1^flox/+^* RIP-Cre
PR: *Pten^flox/flox^* RIP-Cre
P: *Pten^flox/flox^*
MPR: *Men1^flox/flox^ Pten^flox/flox^* RIP-Cre
M”1”PRC: *Men1^flox/+^ Pten^flox/flox^* RIP-Cre
MP”1”RC: *Men1^flox/flox^ Pten^flox/+^* RIP-Cre
M”1”P”1”RC: *Men1^flox/+^ Pten^flox/+^* RIP-Cre
PRbR: *Pten^flox/flox^ Rb1^flox/flox^* RIP-Cre
PRb: *Pten^flox/flox^ Rb1^flox/flox^*
53RbR: *Trp53^flox/flox^ Rb1^flox/flox^* RIP-Cre
53R: *Trp53^flox/flox^* RIP-Cre
53Rb: *Trp53^flox/flox^ Rb1^flox/flox^*
53PR: *Trp53^flox/flox^ Pten^flox/flox^* RIP-Cre
53MR: *Trp53^flox/flox^ Men1^flox/flox^* RIP-Cre
