# Supplementary material for: Genetic analysis of the cooperative tumorigenic effects of targeted deletions of tumor suppressors Rb1, Trp53, Men1, and Pten in neuroendocrine tumors in mice
Source: Oncotarget. 2020 Jul 14;11(28):2718–39. doi: 10.18632/oncotarget.27660 (PMC7367653; doi:10.18632/oncotarget.27660)
Supplement: Supplementary file 1 [file oncotarget-11-2718-s001.pdf]

Genetic analysis of the cooperative tumorigenic effects of targeted deletions of tumor suppressors *Rb1*, *Trp53*, *Men1*, and *Pten* in neuroendocrine tumors in mice

SUPPLEMENTARY MATERIALS

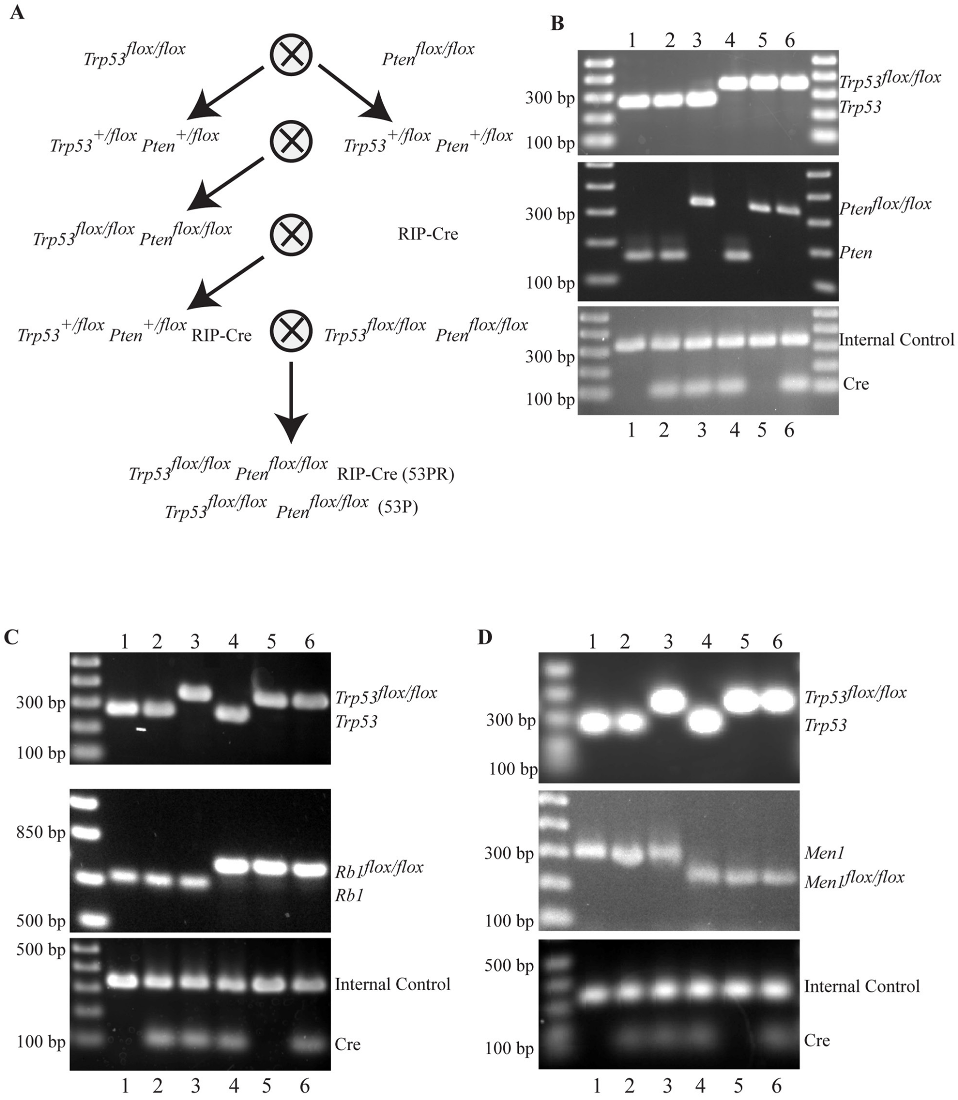

Supplementary Figure 1: Strategies for generating 53PR compound mice and results of PCR analysis. (**A**) Diagram of the strategy used to generate compound mice 53PR and control 53P mice. (**B**) Representative genotyping results of the litters in A by PCR analysis using tail genomic DNA. Genotypes of each lane: 1-WT, 2-RIP-Cre, 3-*Pten*$^{flox/flox}$ RIP-Cre (PR), 4-*Trp53*$^{flox/flox}$ RIP-Cre (53R), 5-*Trp53*$^{flox/flox}$ *Pten*$^{flox/flox}$ (53P), 6- *Trp53*$^{flox/flox}$ *Pten*$^{flox/flox}$ RIP-Cre (53PR). (**C**) Representative genotyping results of the compound mice 53RbR and 53Rb by PCR analysis using tail genomic DNA. Genotypes of each lane: 1-WT, 2-RIP-Cre, 3- *Trp53*$^{flox/flox}$ RIP-Cre (53R), 4-*Rb1*$^{flox/flox}$ RIP-Cre (RbR), 5-*Trp53*$^{flox/flox}$ *Rb1*$^{flox/flox}$ (53Rb), 6- *Trp53*$^{flox/flox}$ *Rb1*$^{flox/flox}$ RIP-Cre (53RbR). (**D**) Representative genotyping results of the compound mice 53MR and 53M by PCR analysis using tail genomic DNA. Genotypes of each lane: 1-WT, 2-RIP-Cre, 3- *Trp53*$^{flox/flox}$ RIP-Cre (53R), 4-*Men1*$^{flox/flox}$ RIP-Cre (MR), 5-*Trp53*$^{flox/flox}$ *Men1*$^{flox/flox}$ (53M), 6- *Trp53*$^{flox/flox}$ *Men1*$^{flox/flox}$ RIP-Cre (53MR).

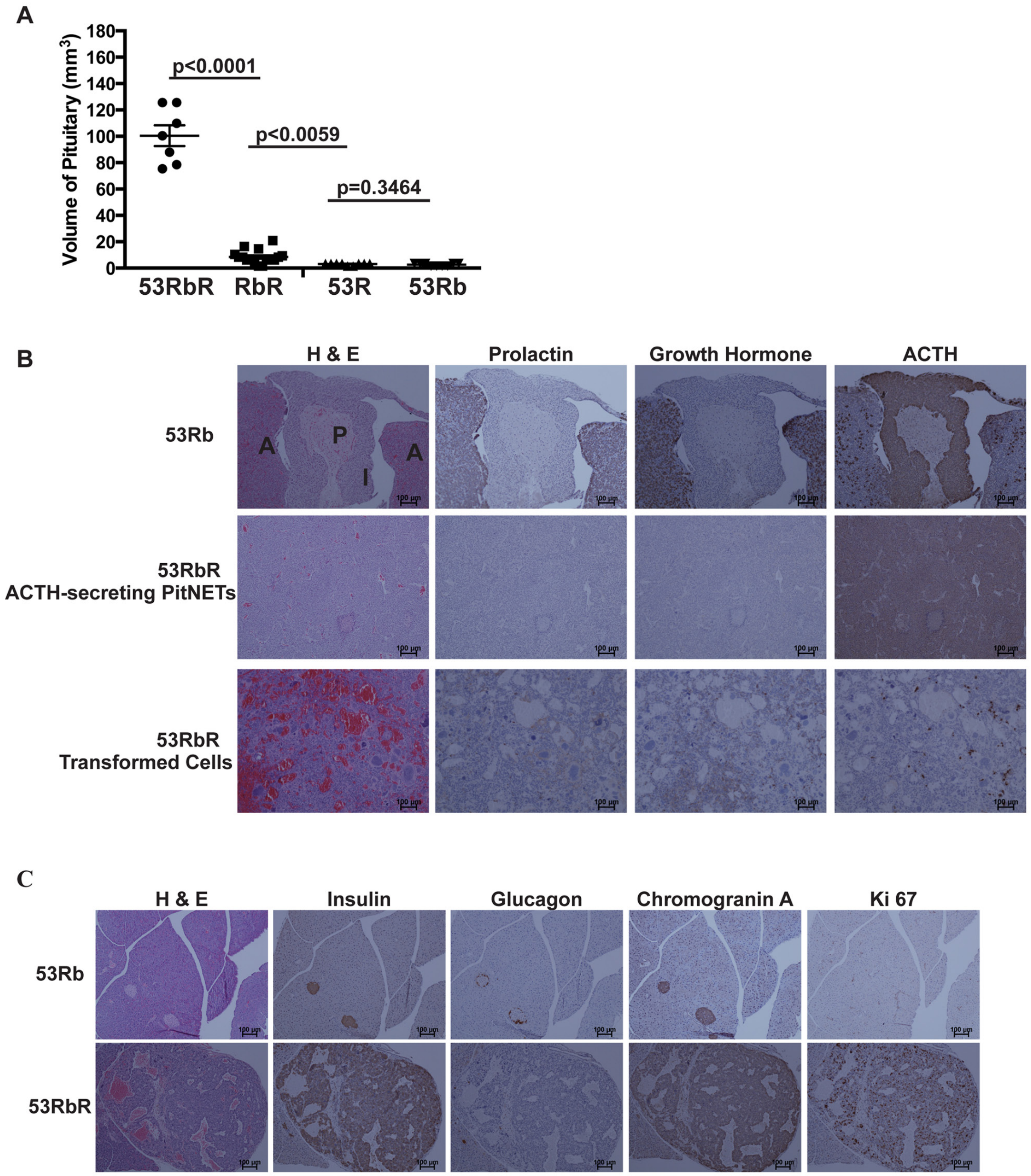

Supplementary Figure 2: *Trp53* and *Rb1* had cooperative function to suppress PitNETs and PanNETs. (**A**) The sizes of PitNETs in 53RbR mice at death were significantly larger than that in single deletion RbR and 53R and wild-type control 53Rb mice of the same age and sex as shown *p*-values. (**B**) H & E, IHC staining of prolactin, growth hormone and ACTH of PitNETs in 53RbR mice showed two types of cells: ACTH-secreting PitNETs (middle panel) and transformed cells (bottom panel) while IHC staining of pituitary in wild-type control 53Rb mice showed normal staining. Anterior lobe (A), Intermediate lobe (I) and Posterior lobe (P) of normal pituitary in 53Rb mice are shown in the H & E section. (**C**) H & E, IHC staining of Insulin, Glucagon, Chromogranin A, and Ki67 of pancreas sections from 53Rb and 53RbR mice. All images were taken with the same magnifications.
